# Variability and stability of large-scale cortical oscillation patterns

**DOI:** 10.1162/netn_a_00046

**Published:** 2018-10-01

**Authors:** Roy Cox, Anna C. Schapiro, Robert Stickgold

**Affiliations:** Department of Psychiatry, Beth Israel Deaconess Medical Center and Harvard Medical School, Boston MA, USA; Department of Psychiatry, Beth Israel Deaconess Medical Center and Harvard Medical School, Boston MA, USA; Department of Psychiatry, Beth Israel Deaconess Medical Center and Harvard Medical School, Boston MA, USA

**Keywords:** EEG, Oscillations, Networks, Functional connectivity, Individual differences

## Abstract

Individual differences in brain organization exist at many spatiotemporal scales and underlie the diversity of human thought and behavior. Oscillatory neural activity is crucial for these processes, but how such rhythms are expressed across the cortex within and across individuals is poorly understood. We conducted a systematic characterization of brain-wide activity across frequency bands and oscillatory features during rest and task execution. We found that oscillatory profiles exhibit sizable group-level similarities, indicating the presence of common templates of oscillatory organization. Nonetheless, well-defined subject-specific network profiles were discernible beyond the structure shared across individuals. These individualized patterns were sufficiently stable to recognize individuals several months later. Moreover, network structure of rhythmic activity varied considerably across distinct oscillatory frequencies and features, indicating the existence of several parallel information processing streams embedded in distributed electrophysiological activity. These findings suggest that network similarity analyses may be useful for understanding the role of large-scale brain oscillations in physiology and behavior.

## Author Summary

Neural oscillations are critical for the human brain’s ability to optimally respond to complex environmental input. However, relatively little is known about the network properties of these oscillatory rhythms. We used electroencephalography (EEG) to analyze large-scale brain wave patterns, focusing on multiple frequency bands and several key features of oscillatory communication. We show that networks defined in this manner are, in fact, distinct, suggesting that EEG activity encompasses multiple, parallel information processing streams. Remarkably, the same networks can be used to uniquely identify individuals over a period of approximately half a year, thus serving as neural fingerprints. These findings indicate that investigating oscillatory dynamics from a network perspective holds considerable promise as a tool to understand human cognition and behavior.

## INTRODUCTION

Although human brains are very similar, every brain is also distinct. Magnetic resonance imaging (MRI) techniques indicate both individual variability in anatomical white matter connectivity (Bürgel et al., 2006) and marked differences in interregional functional connectivity (Gordon, Laumann, Adeyemo, & Petersen, 2015) that relate to cognitive functioning (Finn et al., 2015; Mueller et al., 2013; Schultz & Cole, 2016). In recent years, MRI network approaches have yielded powerful insights into the brain’s macroscopic connectivity pattern, or connectome, and its relation to behavior (Bullmore & Sporns, 2009; van den Heuvel & Sporns, 2013). Unlike MRI, electroencephalographic (EEG) and magnetoencephalographic (MEG) techniques are sensitive to rapid, millisecond fluctuations in the electromagnetic fields generated by neuronal populations, and are therefore more suitable to examine the highly dynamic nature of rhythmic brain activity. Moreover, multichannel EEG combined with spatial filtering techniques offers a reasonable degree of topographical precision, thereby allowing investigation of the “oscillatory connectome”—the pattern of distributed oscillatory interactions across the cortex. Yet, relatively little is known about the detailed properties of such oscillatory networks, their variability from person to person, or their long-term stability.

Distinct brain oscillations underlie specific cognitive functions (Lopes da Silva, 2013; Siegel, Donner, & Engel, 2012; Thut, Miniussi, & Gross, 2012), and specific frequencies are expressed differently across the brain (Congedo, John, De Ridder, & Prichep, 2010; Keitel & Gross, 2016). Moreover, different aspects of rhythmic activity are thought to capture distinct aspects of brain organization and function: whereas oscillatory power reflects the strength of local rhythmic activity in a particular frequency band, functional connectivity assesses temporally coordinated activity between brain areas in a similarly band-specific manner. In particular, consistent phase relations between brain circuits are thought to mediate efficient neural communication on a cycle-by-cycle basis (Fell & Axmacher, 2011; Fries, 2005), whereas coordinated fluctuations of signal amplitude capture slower aspects of interregional communication (Bruns, Eckhorn, Jokeit, & Ebner, 2000) and relate to the correlation structure observed with functional MRI (Hipp & Siegel, 2015). Importantly, these different measures of activity and connectivity can be dissociated (Arnulfo, Hirvonen, Nobili, Palva, & Palva, 2015; Bruns et al., 2000; Hillebrand, Barnes, Bosboom, Berendse, & Stam, 2012), suggesting they reflect distinct facets of neural dynamics (Bastos & Schoffelen, 2016; Cohen, 2014a).

This dissociability of oscillatory metrics and frequencies, along with phenomena of cross-frequency coupling (Aru et al., 2014), has instilled the notion that macroscopic electrophysiological signals reflect multiplexed activity, composed of multiple communication lines operating in parallel (Ainsworth et al., 2012; Akam & Kullmann, 2014; Panzeri, Macke, Gross, & Kayser, 2015; Watrous, Fell, Ekstrom, & Axmacher, 2015). Importantly, such concurrently present signals can serve functionally distinct roles (Gross et al., 2013; Schyns, Thut, & Gross, 2011; Watrous, Tandon, Conner, Pieters, & Ekstrom, 2013) and could constitute a fundamental computational principle to increase information processing capacity. In light of accumulating evidence for the fundamental role of distributed oscillatory activity in neuronal communication (Canolty et al., 2010) and cognition (Honkanen, Rouhinen, Wang, Palva, & Palva, 2015; J. M. Palva, Monto, Kulashekhar, & Palva, 2010), an intriguing possibility is that multiplexing can be discerned at the network level. The coexistence of multiple such network configurations might constitute an important principle of human brain organization, and could offer novel analysis strategies to elucidate brain functioning. However, it is presently unclear whether decomposition of brain-wide oscillation patterns based on frequency or oscillatory metric yields separable activity patterns, nor is it known if and how this dissection depends on behavioral state.

An additional issue regards the variability of oscillatory patterns across individuals. Previous evidence indicates that network structure differs substantially between individuals (Chu et al., 2012), but it is unknown how different frequency bands and oscillatory metrics contribute to this variability. Similarly, spatially organized oscillatory activity remains stable within an individual over multiple days (Chu et al., 2012), but longer-term stability of frequency- and metric-specific networks has not been assessed. Given accumulating evidence that individual differences in rhythmic activity predict cognitive performance (Jiang, van Gerven, & Jensen, 2015; Klimesch, Schimke, Ladurner, & Pfurtscheller, 1990; Park et al., 2014), fine-grained characterization of individual oscillatory network differences and stability is critical for a complete understanding of human brain organization.

Prior work on large-scale oscillatory dynamics (e.g., Brookes et al., 2012, 2014; Chu et al., 2012; Hillebrand et al., 2012; Hipp, Hawellek, Corbetta, Siegel, & Engel, 2012; Keitel & Gross, 2016; Siems, Pape, Hipp, & Siegel, 2016) has provided valuable insights into various of these aspects of network organization. However, individual studies have focused on only one or a few of these features, making it difficult to determine how these various sets of findings interrelate. We set out to systematically characterize the brain-wide structure of oscillatory networks across all of the aforementioned dimensions, comparing EEG network patterns within and between (1) individuals, (2) behavioral states, (3) frequency bands, (4) distinct metrics of spectral power, phase synchrony, and amplitude envelope correlation, and (5) time points hours to months apart. Here, following published approaches to assessing distributed activity patterns (Haxby, Connolly, & Guntupalli, 2014; Kriegeskorte, 2008), we focus on describing global multivariate network structure, as opposed to cataloging the (in)variance of individual network elements across network types. Employing network similarity and classification techniques, we demonstrate the existence of several highly distinct oscillatory profiles operating in parallel, both across and within individuals. Nevertheless, we find within-subject patterns to be sufficiently stable and unique across test sessions spaced hours to months apart to allow successful long-term identification of individual subjects. This comprehensive examination of oscillatory network dynamics facilitates the integration of a diverse set of findings into a single, unified framework of oscillatory human brain organization.

## RESULTS

**Figure 1.**
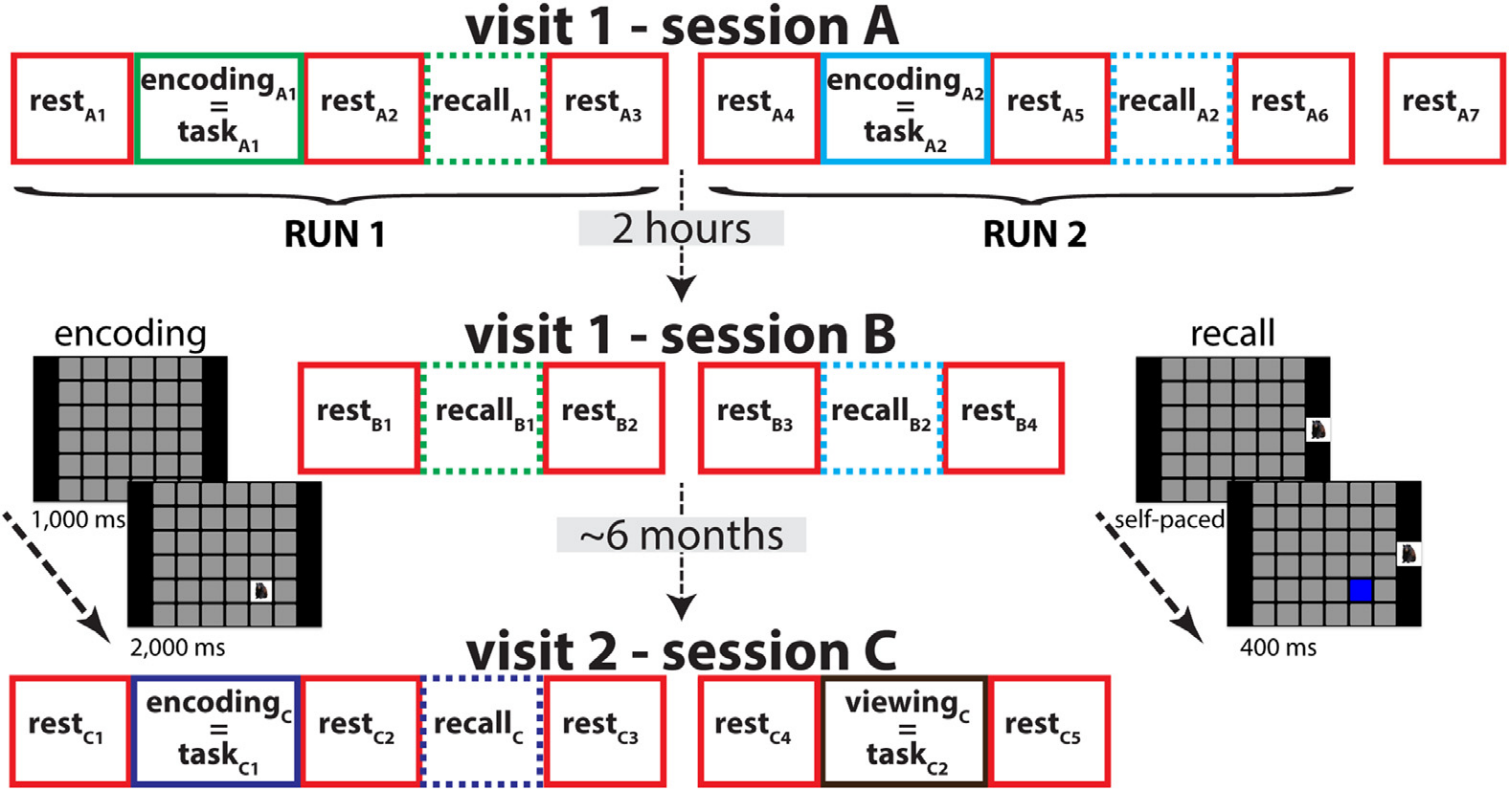
Protocol overview. S_A_ and S_B_ were separated by 2 hr, whereas S_C_ took place after 3–8 months. In S_A_, encoding and recall blocks were separated by rest periods. In S_B_, additional recall blocks were interspersed with rest. Finally, in S_C_, subjects completed an additional memory task as well as a viewing control task with no memory component. EEG from rest and task blocks (solid boxes), but not recall blocks (dashed), was analyzed. During encoding, 36 stimuli were presented, one at a time, at unique grid locations. During retrieval, subjects were cued by presentation of a learned stimulus to the right of the grid, and attempted to identify its previous grid location.

Twenty-one healthy young volunteers completed either one or two visits to the lab (Figure 1). During the first visit, all subjects underwent 60-channel EEG recording during several eyes-closed resting-state blocks organized around the encoding and retrieval of visuospatial associations. The visit included two sessions, separated by 2 hr (Sessions A and B; S_A_ and S_B_). Three to eight months later, 14 subjects returned for a second visit (Session C; S_C_), consisting of several additional resting states recordings, another visuospatial memory task, and a nonlearning control task. In this paper, we focus on the network structure of rest and task (i.e., encoding and control) blocks. Individual blocks are labeled according to their behavioral state, session and order, resulting in a total of 20 blocks across three sessions (S_A_: rest_A1_–rest_A7_ and task_A1_–task_A2_; S_B_: rest_B1_–rest_B4_; S_C_: rest_C1_–rest_C5_ and task_C1_–task_C2_). Memory performance is reported in the Supporting Information (Cox, Schapiro, & Stickgold, 2018).

We collected ∼5-min continuous, surface Laplacian-filtered (Kayser & Tenke, 2006; Perrin, Pernier, Bertrand, & Echallier, 1989) EEG segments from each block of rest and task activity. We then determined spectral power at each electrode and calculated phase synchrony and amplitude envelope correlation for every pair of electrodes (excluding 11% of connections between neighboring electrodes to restrict undesired volume conduction effects). All oscillation metrics (power and both amplitude- and phase-based functional connectivity) were determined separately for the theta (3–7 Hz), alpha (8–12), beta (13–30), and gamma (32–60) frequency bands. For our main analyses we used the phase locking value (Lachaux, Rodriguez, Martinerie, & Varela, 1999) and conventional amplitude correlations (Bruns et al., 2000) as measures of functional connectivity. We also performed several control analyses by using alternative functional connectivity metrics (i.e., weighted phase lag index [Vinck, Oostenveld, Van Wingerden, Battaglia, & Pennartz, 2011] and orthogonalized envelope correlations [Hipp et al., 2012]) to confirm that our results cannot be explained by inflated connectivity estimates resulting from volume conduction (Supporting Information, Cox et al., 2018). For the sake of brevity, we adopt the term “connectivity” as a shorthand for “functional connectivity” throughout this paper.

### Similarity of Large-Scale Oscillatory Networks

**Figure 2.**
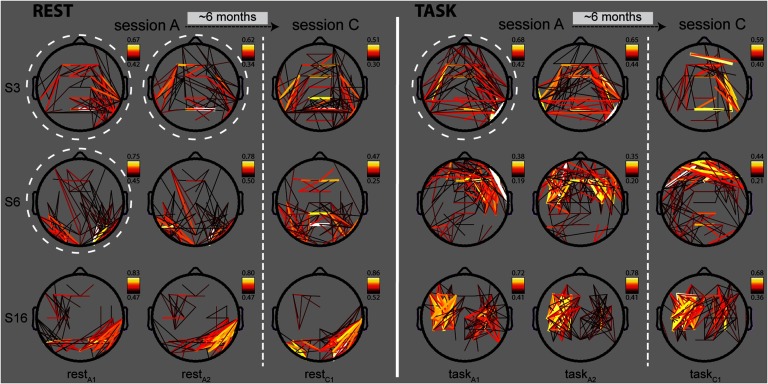
Connectivity maps of phase-based alpha networks. Absolute phase synchrony in the alpha band for three example subjects (rows), during three rest and three task segments across S_A_ and S_C_ (columns). Strength of connectivity is indicated by both line thickness and color, with stronger connections in white/yellow, and weaker connections in orange/black. For visualization purposes, only connections between the median + 2 SD and maximum connection strength are shown for each map (range indicated on color bar). White dashed circles indicate networks used to illustrate network similarity in Figure 3A–C.

We first assessed absolute levels of spectral power and phase- and amplitude-based connectivity for rest and task segments. In brief, global and topographical measures of oscillatory activity varied with frequency and were affected differently by task and rest conditions (Supporting Information Results and Supporting Information Figure S1, Cox et al., 2018). To examine the underlying oscillatory profiles contributing to these group effects, we visualized participants’ patterns of absolute connectivity across sessions. Figure 2 shows three subjects’ phase-based alpha profiles during several rest and task segments in both S_A_ and S_C_. Strikingly, patterns from the same individual demonstrated a visual resemblance not seen between subjects, in both rest and task. Moreover, subject-specific patterns appeared to be stable across the 3–8 month interval between S_A_ and S_C_. Different, but similarly consistent patterns were observed in most subjects, and distinctly discernible individual profiles appeared to exist for different frequencies and connectivity metrics. Topographical power profiles also appeared to be visually stable within subjects, although these effects were less obvious to the naked eye. In addition, whereas rest and task networks typically demonstrated clearly noticeable differences in oscillatory organization, some aspects of individuals’ power and connectivity profiles appeared to be stable across behavioral states. These preliminary visual inspections suggest that distributed patterns of oscillatory activity exhibit an important degree of constancy within subjects, as well as potential differences between behavioral states, frequencies, and oscillatory metrics, prompting quantitative evaluation of these qualitative observations.

A substantial obstacle regarding such analyses is that measures of absolute activity do not allow meaningful comparisons of distributed oscillatory patterns as a function of the aforementioned dimensions, since activity levels are often on different scales or even have different units. These issues can be avoided by comparing the *relative* distribution of oscillatory activity across the cortex and its consistency from one condition to another. Thus, by focusing on network similarity, heterogeneous patterns of oscillatory activity are effectively brought into a common space, enabling direct comparisons across a multitude of dimensions (by individual, behavioral state, frequency band, oscillation metric, and across time).

Throughout this paper, we use the term “network” to refer to vectors that reflect the brain-wide pattern of oscillatory activity across all electrodes or connections. For connectivity, we used vectors of length 1,578 corresponding to every unique channel pair’s connectivity strength (creating separate vectors for each subject, data segment, and frequency-band combination). For spectral power, we constructed vectors of length 60 reflecting all electrodes’ power estimates. In addition, we constructed “power connectivity” vectors of equal size as the connectivity vectors to enable direct comparisons (see Methods). A specific “network type” reflects a category of power/connectivity vectors defined by a particular combination of oscillatory dimensions (e.g., “amplitude-based beta networks during rest”).

**Figure 3.**
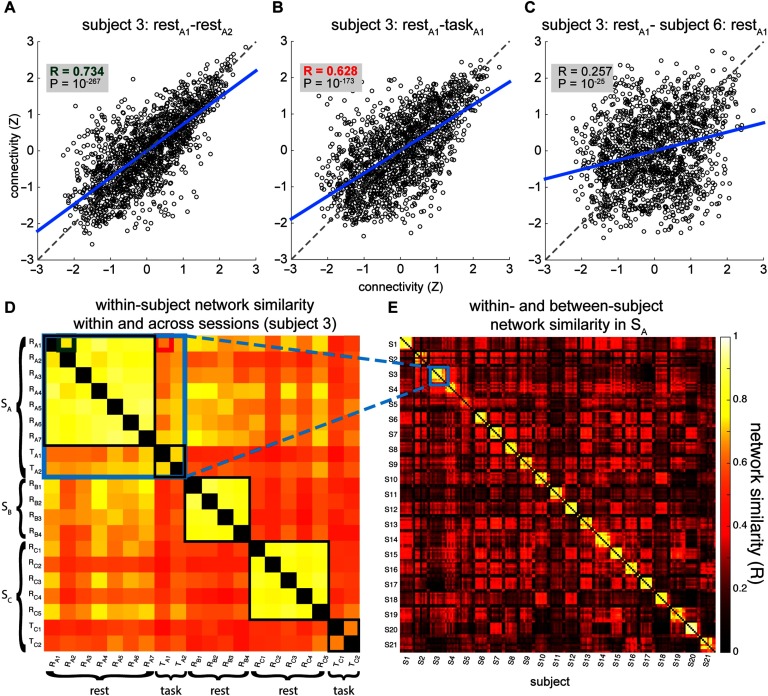
Similarity of phase-based alpha networks. Example scatterplots show network similarity between (A) a single subject’s rest_A1_ and rest_A2_ segments; (B) the same subject’s rest_A1_ and task_A1_ segments; and (C) rest_A1_ from the same subject and the corresponding rest_A1_ of a second subject (selected networks indicated in Figure 2 with dashed circles). Every dot denotes the connection strength between a pair of electrodes (1,578 in total) for two separate data segments: the Pearson correlation coefficient (*R*) constitutes the degree of network similarity. Axes indicate z-scored connectivity strength, and blue lines reflect least-squares fit. Note that as a result of the large number of network elements even modest associations have very low *p* values. (D) Single-subject network similarity matrix of all 20 data segments. Small green and red squares reflect network comparisons of panels A and B, respectively. Large black squares indicate similarity of within-session rest or task networks. Large blue square indicates similarity within S_A_, which is further illustrated in (E), which contains the 7 rest and 2 task S_A_ segments for all subjects. Clearly visible is the diagonal band showing high within-subject similarity. The off-diagonal pattern demonstrates the generally much greater between-subject similarity of rest-rest and task-task networks compared with rest-task networks. Specifically, the larger red/orange squares indicate relatively enhanced between-subject similarity of rest networks, whereas darker bands signify reduced rest-task similarity. Very small red squares positioned on intersecting dark bands indicate increased task-task similarity. For both D and E, diagonal elements (indicating self-similarity) were set to zero.

We quantified the degree of similarity between any two networks (i.e., two vectors) as their Pearson correlation: high similarity between networks indicates a relatively preserved, and therefore consistent, configuration of connection strengths or local power across the scalp, irrespective of possible differences in absolute connection strength or power. Thus, the correlation-based approach critically enables the comparison of heterogeneous network types. Illustrative scatterplots for alpha phase-based networks from one subject demonstrate the generally high correspondence of connection weights between segments derived from the same subject, both within (Figure 3A) and across (Figure 3B) behavioral states. In contrast, network similarity between two different subjects was much lower (Figure 3C).

#### Network consistency within individuals.

To examine the notion of within-subject consistency of network configurations, we assessed the similarity among each subject’s data segments (Kriegeskorte, 2008). This is illustrated for a single subject’s phase-based alpha networks in Figure 3D. Within S_A_, we compared task segments by computing the correlation between task_A1_ and task_A2_, whereas for resting states we computed all 21 pair-wise correlations between a subject’s rest segments (rest_A1_-rest_A7_) and averaging the resulting values. We performed this analysis separately for each of 12 network types (4 frequencies × 3 oscillation metrics). We observed substantial within-subject network similarity, with average Pearson coefficients ranging from 0.49 for theta amplitude correlation during task to 0.98 for alpha power during rest (Supporting Information Table 1A, Cox et al., 2018; due to the large number of network comparisons we performed, here and throughout this report, results are presented at a summary level, whereas detailed network similarity values and statistics are presented in Supporting Information Tables). We also determined each subject’s network consistency between rest and task segments. Here, we calculated, for every subject, the average correlation between each of the 14 unique pairs of rest-task segments (7 rest × 2 task). Compared with the similarity of networks from a single behavioral state, correlations were reduced, but still sizable (range: 0.33 for alpha synchrony to 0.73 for gamma power; Supporting Information Table 1A, Cox et al., 2018). Overall, within-subject similarity scores indicate that network profiles are highly correlated, with strong effect sizes within a behavioral state, and moderate to strong effects between rest and task.

**Figure 4.**
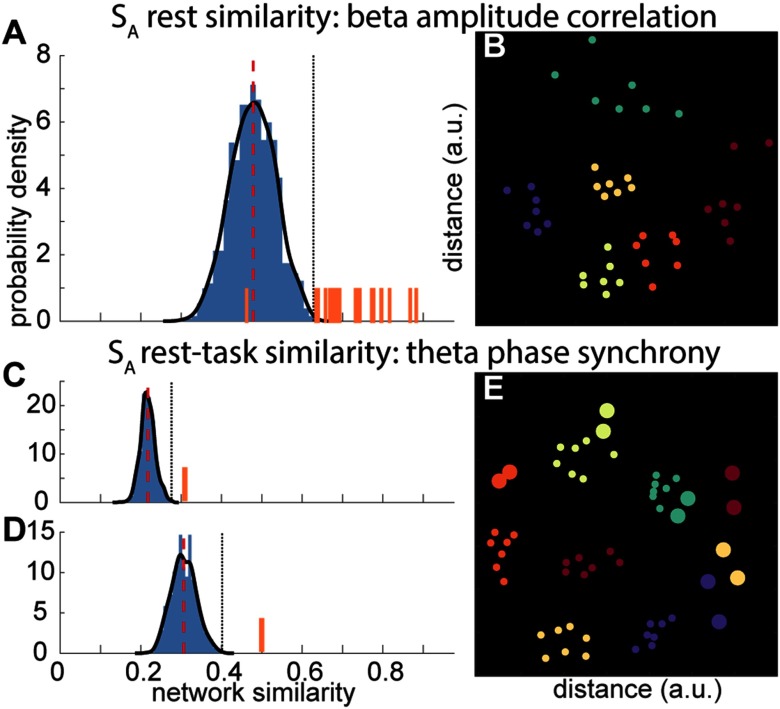
Within-subject similarity of rest and task segments in S_A_. (Top) Similarity (Pearson’s R) of rest segment networks based on amplitude correlation in the beta band. (A) Observed within-subject similarity values (orange bars) are much higher than for the null distribution generated by resampling across subjects (1,000 permutations; dashed red line: mean of null distribution; dotted black line: maximum value in distribution). (B) Multidimensional scaling plot shows similarity between networks for same network type as in A as distances between dots, using the correlation distance (1–R) as the distance metric. Each color represents a single individual. Dots of the same color are generally clustered together, reflecting high intraindividual network similarity. For visualization purposes only 6 subjects are plotted, although clustering is equally present when including all 21 subjects. (Bottom) Similarity between task and rest segments for theta phase synchrony networks. Each subject’s similarity score across behavioral states was compared with its own null distribution (created by assessing network similarity between that subject’s task segments and rest segments randomly selected from the entire population). Distributions for two subjects (C and D) show much higher within-subject similarity between rest and task structure (orange bars) than expected by chance (1,000 permutations). (E) Distance plot for rest-task similarity, as presented in C and D. Smaller dots indicate rest networks (as above) and larger dots signify task networks. For several subjects, their two task segments are close to their seven rest segments, indicating a close correspondence between network structures across behavioral states. At the same time, task networks from different subjects tend to cluster together to the right of the plot, suggesting group-level differences between task and rest networks. Again, only six subjects are plotted for visualization purposes.

These within-subject correlations reflected network similarities greater than those seen between subjects, as can be seen qualitatively from the network similarity matrix in Figure 3E. To demonstrate this quantitatively, we adopted a resampling approach in which we randomly selected networks from the pool of all subjects. Keeping network type constant, we repeatedly shuffled subject labels to generate a null distribution of similarity values (see Methods). Null distributions for rest-rest and rest-task comparisons are shown in Figure 4A, C, and D. Individual subjects’ values (orange bars) had far higher similarity values than expected by chance, and were often the most extreme scores. As a complementary tool, we employed multidimensional scaling techniques to visualize the relatedness of these networks (Figure 4B and E). These plots, with each subject coded in a separate color, demonstrate that oscillatory profiles for the seven rest and two task segments from one individual are often tightly clustered together in multivariate space, indicating that network structure is highly stable for a given individual.

This apparent within-subject stability of oscillatory networks was confirmed in two ways. First, at the group level, we performed a series of 12 one-sample *t* tests (one per network type) comparing the distribution of observed similarity scores across subjects to a null hypothesis baseline defined as the average similarity across permutations (e.g., Figure 4A, C, and D: dashed red lines in the centers of the null distributions). For all network types and data segment comparisons (rest-rest, task-task, and rest-task) this yielded highly significant results (all *p* < 0.002; Supporting Information Table 1A, Cox et al., 2018: group *t* test), indicating that S_A_ oscillatory profiles are significantly more similar within than between subjects.

Within-subject network stability was confirmed at the single-subject level as well. To show this, we z-scored subjects’ network similarity estimates with respect to their own null distributions and calculated the associated *p* values. We then used the false discovery rate (Benjamini & Hochberg, 1995) to correct for multiple tests across individuals (Supporting Information Table 1A, Cox et al., 2018; individual permutation). Depending on network type, between 90.5% (e.g., gamma phase synchrony) and 100% (e.g., beta power) of individual subjects displayed significant network similarity (P_corr_ < 0.05) within their resting-state recordings. For task segments, within-subject network stability was significant for 57.1–90.5% of subjects across network types, except for beta and gamma power profiles (where no subjects showed significant network stability). Finally, for rest-task similarity 66.7–100% of subjects exhibited significant network stability across these behavioral states.

We note that when more lenient uncorrected thresholds were applied to assess significance of task-task similarity, there was significant network stability in the majority of subjects for all network types, and in more than 70% of subjects for 10 of the 12 types. Still, subject proportions showing significant within-subject network similarity at this more lenient threshold were significantly higher for rest-rest than for task-task comparisons across network types (96.4 ± 4.1% vs. 76.6 ± 10.7%; paired *t* test *t*[11] = 6.7, *p* < 10^−4^). However, when we repeated these analyses using only two rest segments (rest_A2_ and rest_A5_), matching the number we had available for task segments, subject proportions showing significant rest-rest similarity dropped significantly to 79.0 ± 17.0% (*t*[11] = –4.2, *p* = 0.002; Supporting Information Table 1B, Cox et al., 2018) and no longer differed appreciably from task-task comparisons (*t*[11] = 0.6, *p* = 0.56). These findings indicate that the number of available networks is a relevant factor when assessing significance in a permutation framework, although we note that this did not affect the observed network similarity values themselves (paired *t* test across network types based on seven [0.75 ± 0.12] or two [0.75 ± 0.12] rest segments: *t*[11] = −1.7, *p* = 0.11).

Finally, we replicated the results in this section for the second session of the first visit (S_B_) and for the second visit (S_C_) up to 8 months later (Supporting Information Table 1C and D, Cox et al., 2018), providing independent confirmation of within-subject network consistency within a single recording session.

#### Distinct rest and task network profiles across individuals.

Analyses of network patterns showed striking differences between rest and task behavioral states. This can be seen from the off-diagonal checkered structure of Figure 3E, indicating that different subjects’ rest segments, and separately, different subjects’ task segments, were more similar to each other compared with networks similarity between behavioral states. Similarly, in the distance plot displayed above (Figure 4E), task segments (larger dots in the lower right of the plot) from different subjects appeared to cluster together, suggesting group-level differences between task and rest network structures. To investigate this further, we calculated group-level similarity across all subjects’ rest segments, and separately, across all task segments, and then compared these values with a baseline distribution of similarity scores obtained through resampling from the combined pool of rest and task segments across subjects. Calculations were carried out separately for each network type.

Observed similarity scores during S_A_ varied depending on network type analyzed (Supporting Information Table 2A, Cox et al., 2018), but overall, 10/12 network types exhibited network configurations that were significantly clustered in multivariate space for rest, task, or both behavioral states. These findings indicate that network organization across individuals within a behavioral state (rest, task) is more similar than would be expected by chance, confirming the visual impression from Figures 3 and 4. We repeated this procedure for the rest and task segments from S_C_ and obtained similar results (Supporting Information Table 2B, Cox et al., 2018). Thus, in addition to individual differences in network organization, both rest and task networks share common power and connectivity profiles across subjects.

We asked whether the observed group-level rest-task differences would allow us to predict behavioral state from network structure by using a supervised learning strategy. For each network type, we trained a *k*-nearest neighbors classifier (Cover & Hart, 1967) on S_A_ rest and task networks from all subjects. In a cross-validated approach, we repeatedly left out each subject’s networks from the training procedure and allowed the classifiers to predict their associated behavioral state. We obtained significantly greater than chance (50%) performance for all 12 network types (binomial tests: all P_corr_ < 0.04). Recognition rates ranged from 59.5% for amplitude- and power-based gamma networks, to 88.1% for phase- and amplitude-based alpha networks (Supporting Information Table 2A, Cox et al., 2018). Average performance across the 12 classifiers (i.e., network types) was 77 ± 10%. Rest networks were more accurately classified than task patterns (83 ± 19% vs. 71 ± 12%), although the difference was not significant (*t*[11] = 1.85, *p* = 0.09). Merging evidence from individual classifiers, each based on a different network type (see Methods), we obtained a classification rate of 92%, indicating different network types are sensitive to different aspects of rest-task differences. Repeating these analyses for S_C_, we again found considerable evidence for distinct task and rest-based networks (Supporting Information Table 2B, Cox et al., 2018).

We next asked whether rest-task similarity *within* individuals was greater than the similarity of each of these behavioral states *across* individuals. Across network types, we found that rest and task networks from the same individual were more similar to each other than rest networks selected across individuals (S_A_: 0.53 ± 0.13 vs. 0.47 ± 0.15; *t*[11] = 2.6, *p* = 0.02; S_C_: 0.56 ± 0.12 vs. 0.43 ± 0.14; *t*[11] = 9.4, *p* < 10^−5^). For individual network types, we found consistent significant effects across S_A_ and S_C_ for all phase-based networks, for amplitude networks in the beta and gamma ranges, and for theta and gamma power profiles (Supporting Information Table 3A and B, Cox et al., 2018). Repeating these analyses for task networks, we found that rest and task networks from the same individual were also significantly more similar than task networks from different individuals for S_C_ (0.56 ± 0.12 vs. 0.39 ± 0.12; *t*[11] = 8.5, *p* < 10^−5^), but less robustly so for S_A_ (0.53 ± 0.13 vs. 0.47 ± 0.11; *t*[11] = 1.5, *p* = 0.15). In line with these weaker effects for S_A_, we observed that only beta amplitude networks and theta and alpha power networks showed consistent significant effects across S_A_ and S_C_.

In sum, these observations, together with those in the previous section, demonstrate, first, that, within a session, subject-specific networks are similar between periods of rest and task execution, second, that the oscillatory profiles of these two behavioral states nonetheless exhibit global differences discernible at the group level, and third, that state-invariant subject-specific neural signatures are typically stronger than state-dependent group profiles.

#### Frequency-specific networks for individuals.

The analyses presented above demonstrate reliable within-subject network consistency for all examined frequency bands. However, this leaves unanswered whether oscillatory profiles are similar across frequencies, which would suggest that they derive from the same intrinsic network activity, or whether distinct spectral bands are independently organized, suggesting the existence of multiple parallel modes of neural processing.

**Figure 5.**
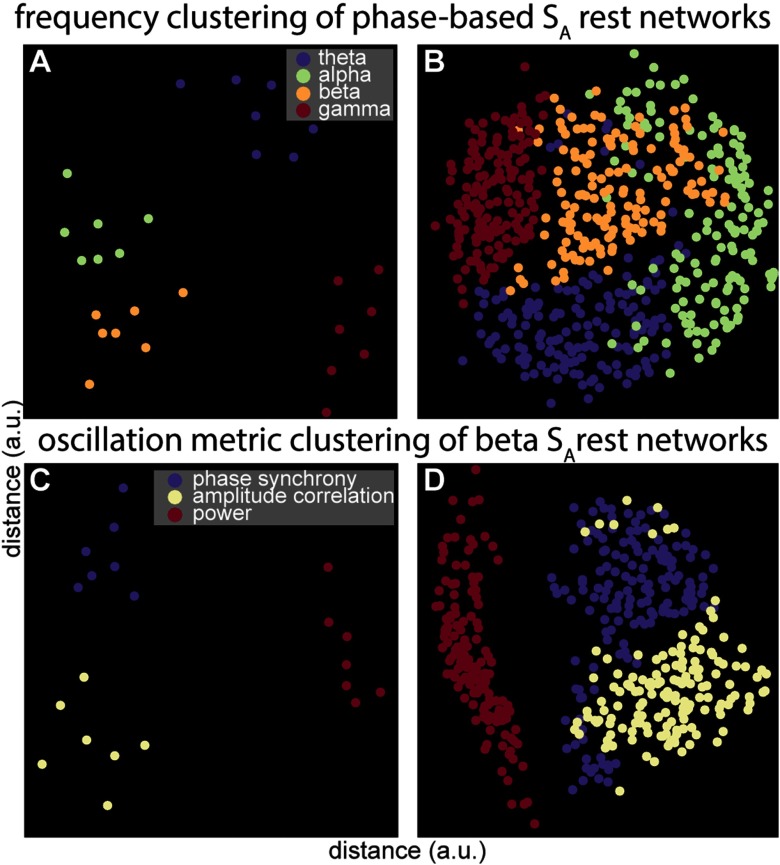
Frequency- and oscillation metric-specific clustering of S_A_ resting-state networks. Single-subject-level (A) and group-level (B) frequency clustering of phase-based networks indicating greater similarity of within-frequency than between-frequency oscillatory profiles. Single-subject-level (C) and group-level (D) oscillation metric clustering in the beta range. Note how power topographies are distinctly different from both phase- and amplitude-based network profiles.

To answer this question, we compared, separately for each individual, the similarity across networks for one frequency band with the similarity seen when comparing networks selected randomly from all four bands. For S_A_ resting states, every subject showed significantly enhanced network similarity within at least one frequency band relative to cross-band similarity (Supporting Information Table 4A, Cox et al., 2018), indicating that the involved frequency-specific networks differed reliably from each other. We found this to be the case for all three oscillation metrics. In terms of frequency bands, networks in the alpha range were most distinctly clustered in multivariate space (for all oscillation metrics), and, in terms of oscillatory feature, networks based on phase synchrony showed most reliable between-frequency differences. Overall, across oscillation metrics and frequency bands, 95.2 ± 8.9% of subjects showed significantly greater than chance within-frequency consistency (Supporting Information Table 4A, Cox et al., 2018; *individual*). We replicated this pattern of results in S_B_ (83.3 ± 15.3%) and S_C_ (79.8 ± 19.5%) (Supporting Information Table 4B and C, Cox et al., 2018). To assist interpretation, Figure 5A employs multidimensional scaling to visualize within-frequency clustering for a sample subject’s phase-based rest segments, where different colors indicate different frequencies.

We performed an analogous set of analyses on each individual’s task segments, for both S_A_ and S_C_. The observed within-frequency similarity was often the most extreme score of all possible permutations, although subject proportions reaching significance across all metrics and frequency bands was reduced relative to rest (S_A_: 31.3 ± 18.0%; S_C_: 32.1 ± 22.1%) (Supporting Information Table 4A and C, Cox et al., 2018). Still, depending on oscillation metric and session, 65–90% of subjects exhibited frequency-specific task networks for at least one frequency. All told, these findings strongly indicate that connectivity and power profiles differ across frequencies within individuals, during both rest and task, suggesting large-scale oscillatory activity is organized in a frequency-specific manner.

#### Frequency-specific networks across individuals.

This frequency-band specificity extends to the group level. Using our permutation approach, we observed significantly enhanced network similarity across subjects within frequency bands for all oscillation metrics during rest segments from S_A_ (Supporting Information Table 4A, Cox et al., 2018: *group*). Correspondingly strong clustering was visible in multidimensional scaling plots (Figure 5B). Similar group-level correspondences were found for S_B_ and S_C_ rest segments (Supporting Information Table 4B and C, Cox et al., 2018). Task segments from S_A_ and S_C_ showed comparable levels of group-level clustering in the beta and gamma bands, but not for theta and alpha connectivity networks (Supporting Information Table 4A and C, Cox et al., 2018). Thus, these findings indicate not only the existence of within-subject, frequency-specific networks, but also the presence of canonical frequency-dependent networks across subjects.

#### Distinct power-, phase-, and amplitude-based networks for individuals.

We also observed important distinctions among networks based on the oscillation metric employed. As stated in the Introduction, estimates of power, amplitude correlation, and phase synchrony are thought to be sensitive to distinct facets of oscillatory activity and communication (Arnulfo et al., 2015; Bastos & Schoffelen, 2016; Bruns et al., 2000; Cohen, 2014a; Hillebrand et al., 2012). However, whether this separation extends to the level of brain-wide EEG patterns is an open question. We asked whether network configurations derived from a single oscillatory metric were reliably more similar than when the networks were randomly selected across oscillatory measures.

Within individuals, within-metric correlation values for S_A_ rest networks were greater than the average correlation stemming from permuting across oscillatory metrics (Supporting Information Table 5A, Cox et al., 2018). All subjects displayed significant within-metric clustering for all oscillation metrics for the theta, beta, and gamma bands, and >90% showed significant clustering for all metrics in the alpha band. Figure 5C displays a corresponding distance plot for a single subject’s resting beta networks. We replicated this pattern of results for S_B_ and S_C_ (Supporting Information Table 4B and C, Cox et al., 2018) with 92.5 ± 8.2% and 91.7 ± 8.0% of subjects, respectively, showing clustering across frequency bands and metrics. (We could not assess the existence of metric-specific networks within individuals for task networks because of the low number of possible permutations; see Methods.) Thus, these findings demonstrate that oscillatory profiles based on different oscillatory features are reliably distinct, even when derived from the same frequency band, for almost all individuals.

#### Distinct power-, phase-, and amplitude-based networks across individuals.

Next, we asked whether, for a given frequency band, networks based on different oscillation metrics are consistently distinct across subjects. Permutation testing demonstrated this to be the case for all frequencies except alpha, for both rest and task segments, for all metrics, and during all sessions. For alpha, at least one metric failed to reach significance in each session (Supporting Information Table 5A–C, Cox et al., 2018). Figure 5D displays the group-level similarity of resting beta networks across oscillation metrics during S_A_. In summary, these analyses demonstrate that power-, phase-, and amplitude-based network patterns are differently organized, not only within but also across individuals.

#### Distinct phase- and amplitude-based networks.

As seen in Figure 5C and D, power networks differed substantially from connectivity networks in general, with phase and amplitude showing less difference. We therefore repeated the preceding subject- and group-level analyses excluding power networks. Within subjects we found that 59.5 ± 26.1%, 48.8 ± 26.3, and 58.0 ± 26.6% of subjects showed significant within-metric clustering across metrics and frequency bands for S_A_, S_B_, and S_C_, respectively (Supporting Information Table 5D–F, Cox et al., 2018). However, 80–100% of individuals showed significant clustering in at least one of the two connectivity metrics for the theta, alpha, and beta bands, and 30–60% for gamma. Generally, phase synchrony networks showed more reliable within-metric network consistency than amplitude-based networks at the individual level. In contrast, group-level network consistency was significant mostly for amplitude-based networks. These effects again occurred for both rest and task networks, and across all sessions and frequency bands. Thus, direct comparisons between functional connectivity networks that are based on mathematically and theoretically distinct measures of neural communication confirm the distinctiveness of these networks, both across and within individuals.

### Long-Term Stability of Large-Scale Oscillatory Networks

#### Network similarity across time.

Although the foregoing analyses indicated high within-subject network consistency within a single 1-h recording session, they do not address the question of longer-term network stability. Using permutation analyses, we asked whether network similarity across sessions was greater within than across individuals, and found substantial evidence that this was the case, across all network types and behavioral states (Figure 2, Supporting Information Table 6A–C, Cox et al., 2018). Indeed, we found very little difference in network similarity across short- (2 hr: S_A–B_) and long-term intervals (3–8 months: S_AB–C_), indicating remarkable stability in oscillatory network organization across time intervals approaching one year (Supporting Information Results, Cox et al., 2018).

#### Long-term classification of data segments.

Based on this striking network stability over time, we asked whether a supervised learning technique might allow long-term identification of individuals. To address this question, we trained a set of *k*-nearest neighbors classifiers, one for each network type, on the combined S_A_ and S_B_ network configurations. We then used these trained classifiers to predict subject identities of S_C_ networks. Of note, although data of only two-thirds of the original volunteers was available to assess classification accuracy, each classifier was trained on, and allowed to predict, all 21 identities.

After training on all S_AB_ rest or task segments, classifier performance across S_C_ segments was significantly above the chance rate of 4.8% (1/21) for all network types (binomial: all *p* < 10^−10^; permutation: all *p* < 0.001; Table 1). On the whole, the set of rest classifiers performed similarly to the set of task classifiers (*t*[11] = 1.7, *p* = 0.12). A control analysis indicated reduced but still highly significant performance with only two data segments per subject used for training and testing (Supporting Information Results, Supporting Information Table 7, Cox et al., 2018). In sum, these findings demonstrate that oscillatory network patterns carry substantial information for classification of individuals across months, for all oscillation metrics and frequency bands, and for periods of both rest and task.

**Table 1.** Classifier performance for all oscillation metrics, frequency bands, and behavioral states. Numbers indicate percentage of data segments correctly identified. All classifiers performed significantly above chance (4.8%). *Improved classifier performance when combining frequencies or oscillation metrics. **Further improved performance when combining frequencies and oscillation metrics.

Rest_AB_–rest_C_	*Theta*	*Alpha*	*Beta*	*Gamma*	*Combined across frequencies*
*Phase synchrony*	58.6	72.9	67.1	47.1	75.7*
*Amplitude correlation*	32.9	62.9	62.9	50	68.6*
*Power*	57.1	65.7	54.3	50	71.4*
*Combined across oscillation metrics*	61.4*	81.4*	70.0*	62.9*	81.4

Task_A_–task_C_					
*Phase synchrony*	53.6	78.6	57.1	42.9	78.6
*Amplitude correlation*	35.7	71.4	39.3	57.1	64.3
*Power*	50	42.9	53.6	35.7	53.6
*Combined across oscillation metrics*	57.1*	78.6	75.0*	57.1	82.1**

Our earlier findings highlighted network variability not only across subjects, but also across network types *within* an individual. Thus, combining different classifiers sensitive to partly nonoverlapping information should in theory improve performance. In separate approaches, we fused classifiers across frequency bands, oscillation metrics, or both, separately for rest and task. Combining information across frequency bands numerically improved performance for all oscillation metrics during rest, with each composite classifier showing greater accuracy than the best-performing individual classifier on which it was based (Table 1), although similar improvements were not seen for task segment classification. Combining information across oscillation metrics improved classification accuracy for rest segments in all frequency bands, and, for task segments, in two out of four bands. Finally, when we combined all classifiers, performance was further boosted to 81 and 82% for rest and task segments, respectively, correctly identifying the source of 57 out of 70 rest segments and 23 of 28 task segments (binomial: both *p* < 10^−16^; permutation: both *p* < 0.001). The improved classifier performance observed after merging individual classifiers supports the argument that networks based on different metrics and frequency bands contain unique identifying information.

#### Long-term subject recognition.

Successful subject identification does not require correct classification of each individual data segment. Pooling across an individual’s segments, separately for rest and task segments, classifiers correctly identified 13 of 14 subjects (93%) based on rest networks, and 11 of 14 (79%) using task networks (binomial: both *p* < 10^−13^; permutation: both *p* < 0.001). Task-based classification rates were similar for individual data segments and for subject identity (82% vs. 79%), but the greater number of rest segments available for analysis led to numerically improved subject recognition (93%) relative to data segment classification (81%). In a final step, we also combined rest and task information. Using this approach, we reached perfect accuracy, correctly identifying all 14 subjects (binomial test: *p* < 10^−16^; permutation test: *p* < 0.001).

#### Contribution of individual network types.

We examined the contribution of different network types to subject recognition performance by repeatedly excluding one or more network types from the classifier merger procedure (but retaining both rest and task networks). Removing either all phase- or amplitude-based information (while keeping all frequency bands) did not affect performance, but excluding power topographies decreased classification to 86%. Including only a single oscillation metric, subject recognition was 79% for phase-based networks, and 71% for both amplitude-based networks and power topographies.

Including only single frequency bands (but retaining all oscillation metrics), we obtained classification rates of 64% for theta, 86% for beta, and 79% for gamma. Impressively, including only the alpha band left accuracy at 100%. Using only single oscillation metrics for alpha networks, but still combining rest and task information, resulted in recognition rates of 86% for phase-based, 79% for amplitude-based, and 71% for power-based classifiers. Thus, although alpha activity affords sufficient discriminatory power on its own, alpha networks based on different oscillatory metrics capitalize on different sources of discerning information. However, excluding alpha-based networks altogether still resulted in performance of 86%, indicating that alpha activity is not the sole driver of long-term subject recognition. Excluding only theta, beta, or gamma activity left classifier performance at 93, 100, and 100%, respectively.

Additional analyses revealed that subject recognition was little influenced by parameter settings or the number of segments used for training or classifying individuals (Supporting Information Results, Cox et al., 2018). These findings provide additional evidence that oscillatory profiles from distinct behavioral states, frequency bands, and oscillation metrics act as complementary brain-based fingerprints, each carrying unique identifying information.

#### Impact of network size.

**Figure 6.**
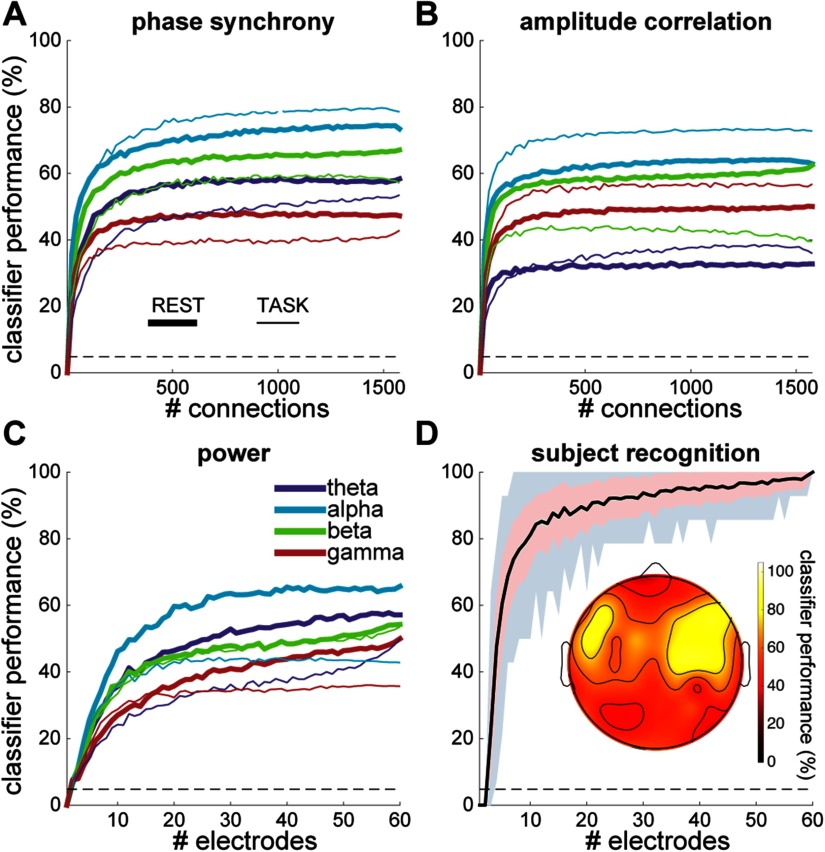
Data segment classification and subject recognition accuracy as a function of number of included connections and electrodes. Percentage of data segments accurately classified as a function of number of included connections for rest and task segments in different frequency bands, for phase synchrony (A) and amplitude correlation (B). For visualization purposes, A and B data were smoothed with a moving average window of size 11 and downsampled by a factor 21. Dashed gray lines indicate chance level performance. (C) Similar to A and B for power as a function of number of included electrodes. (D) Subject recognition as a function of electrode array size (electrodes plus connections among them), including all oscillation metrics, frequency bands, and behavioral states. Black line indicates average, pink shading standard deviation, and gray shading range of minimum and maximum values across 100 iterations. Inset: topographical map displaying subject recognition for searchlight analysis.

To examine how many network elements are required for accurate classification, we varied the number of included elements in each vector (i.e., electrodes or connections) used for training and testing classifiers between two and the maximum number available. Electrodes or connections were selected randomly at each network size, and the entire process was repeated 10 times. For individual data segments, the percentage of networks accurately identified by classifiers trained on a single network type reached a plateau quite early on, when approximately 200 (13%) or 100 (6%) out of 1,578 connections were included for phase- and amplitude-based networks, respectively (Figure 6A and B). Significantly above-chance performance (*p* < 0.05 for one-sample *t* tests comparing each sample of 10 scores to 4.8%), was achieved with as few as 4.2 ± 1.3 connections across different network types, with alpha amplitude correlation showing significant classification using just two connections (rest: 8.9%, *p* = 0.001; task: 7.9%, *p* = 0.03). For power, performance reached stability once 20 (33%) of the 60 electrodes were included (Figure 6C), but significantly higher than chance performance was observed with only two electrodes for all frequency bands and during both rest and task execution (mean: 6.8 ± 1.1%).

Next, we asked how subject recognition rates (i.e., when multiple network types and data segments from the same individual are pooled) depend on these numbers. We randomly selected 2 to 60 electrodes and all pair-wise connections, except neighbors, among them. We repeated this process 100 times for each montage size, training, testing, and combining the different classifiers to assess subject identity for every montage size. Results indicated improved performance with larger electrode arrays, with a shape roughly following that of individual classifiers (Figure 6D). Arrays of 5, 10, and 21 randomly selected electrodes were sufficient to obtain average subject identification rates of 60, 80, and 90%, respectively.

These analyses provide no information as to whether particular clusters of adjacent sensors contribute more to classifier success than others. We performed a searchlight analysis in which, for each electrode, we selected all surrounding electrodes and connections, excluding connections between direct neighbors, within a small radius. We then trained and tested classifiers on subnetworks containing, on average, eight neighboring electrodes (range: 6–11) around each searchlight center, integrating information across all network types and behavioral states. Average subject recognition rate across all searchlight centers was 58 ± 14% (range: 36–86%). When we compared searchlight-based recognition rates to recognition scores from random, and therefore generally more distributed, electrode arrays of similar size (9 electrodes), we observed far superior performance for these distributed networks (79 ± 11%, *t*[158] = 10.2, *p* < 10^−16^). Topographically, searchlight-based performance was highest at 86% in two symmetrically lateralized frontocentral clusters, centered on five electrodes in total (Figure 6D, inset), suggesting that the largest individual differences manifested in these regions. This score was significantly elevated compared with randomly distributed networks of the same size (one-sample *t* test: *t*[99] = 6.2, *p* = 10^−8^). However, topographical peaks for the performance of individual classifiers—based on single network types—were widely distributed across the cortex.

In sum, although greater numbers of included connections and electrodes improve subject recognition rates, a remarkable amount of identifying information can be extracted from networks of much smaller size, especially when electrodes are widely spaced or restricted to frontocentral regions.

## DISCUSSION

The present work offers a systematic analysis of the large-scale network structure of continuous rhythmic brain activity across the scalp. Employing a data-driven approach with internal replications, we have demonstrated that oscillatory network patterns differ across frequency bands and oscillation metrics, suggesting that distinct network types, defined by these parameters, capture separate processing streams operating in parallel. This phenomenon was present across behavioral states of task and rest, which themselves differed robustly in terms of network organization. Moreover, despite clear commonalities in oscillatory patterns across subjects, we also observed prominent individual differences. These individual differences in network profiles were sufficiently stable to allow successful long-term identification of individuals across several months, suggesting that individuals exhibit unique and stable oscillatory fingerprints.

### Multiplexing of Oscillatory Networks

The critical role of neural oscillations and their interactions for cognition is widely recognized (Lopes da Silva, 2013; Siegel et al., 2012; Thut et al., 2012), and is generally thought to result from oscillations transiently and flexibly routing information flow among behaviorally relevant neuronal populations. However, precisely how these dynamics are implemented is a topic of much debate, and numerous schemes have been proposed for how frequency, phase, and amplitude coordinate spiking activity among cell assemblies (Ainsworth et al., 2012; Akam & Kullmann, 2014; Canolty et al., 2010; Fries, 2005; Panzeri et al., 2015; Thut et al., 2012; Watrous et al., 2015). Evidence indicates that multiple coding mechanisms can operate in parallel, simultaneously encoding multiple stimulus attributes at different frequencies and/or by orthogonal phase and amplitude features (Gross et al., 2013; Schyns et al., 2011; Watrous et al., 2013). Our findings advance this notion of multiplexing by decomposing brain-wide EEG activity into statistically separable power-, phase-, and amplitude-based networks, which, in turn, are composed of distinct frequency-specific network configurations. These layered networks can be discerned during periods of both rest and task, and are statistically segregated even within individuals, emphasizing the robustness of these dynamics.

Several previous MEG and EEG studies have identified frequency-specific networks, most commonly by parcellating neural regions and connections into spatially restricted subnetworks (Brookes et al., 2014; Congedo et al., 2010; Hillebrand et al., 2012; Hipp et al., 2012; Keitel & Gross, 2016; J. M. Palva et al., 2010; Siems et al., 2016). But to the best of our knowledge, direct comparisons of the topology of the resulting networks have not been performed. Similarly, previous examinations of power-, phase-, and amplitude-based activity have not addressed these dynamics at the network level. The conclusion that parallel network configurations exist in tandem can only be made after bringing these various network types into a common reference frame and explicitly assessing their similarity, as we have done here.

Our findings of network separability both confirm previous findings and reveal novel insights. Given the well-known posterior distribution of resting alpha activity (Supporting Information Figure 1A, Cox et al., 2018), it is perhaps to be expected that alpha power patterns express high similarity across participants, and that these patterns are distinct from, for example, theta profiles. In contrast, it is not immediately evident that connectivity patterns derived from phase synchrony should differ systematically between, say, the beta and gamma bands. Although such findings may not be deemed surprising in light of known differences between beta and gamma power topographies, they should still be demonstrated rather than assumed. Similarly, given that direct comparisons of power and functional connectivity based on phase and amplitude have been scarce in prior analyses of human physiological recordings (Arnulfo et al., 2015; Bruns et al., 2000; Hillebrand et al., 2012), we believe our findings similarly add important empirical support for the notion that these measures are indeed sensitive to distinct facets of oscillatory dynamics, and furthermore, that these distinctions hold at the network level. Perhaps most importantly, all these frequency- and metric-specific networks can be extracted from the same data in the majority of individuals. We suggest that this organizational principle offers novel opportunities to probe the functional and computational processes underlying cognition from a network perspective.

### State-Dependent Network Organization

Group-level network structure differed significantly between rest and task. This observation is consistent with well-known findings of state-dependent differences in power topographies (e.g., Pfurtscheller, 1992), which we also observed (Supporting Information Figure 1A, Cox et al., 2018). More recent examinations have turned to task-induced changes in functional connectivity (Bassett, Meyer-Lindenberg, Achard, Duke, & Bullmore, 2006; Brookes et al., 2012; Keitel & Gross, 2016). In line with these observations, we found differential rest-task network configurations in various frequency bands as measured by phase synchrony and amplitude correlation structure. Indeed, the demonstration that classifiers could successfully differentiate between an out-of-sample subject’s rest and task states, and, moreover, could do so for all frequency bands and oscillation metrics, further underscores the similarity of network patterns across subjects, as well as the differences between these behavioral states. We again note that although rest-task differences are to be expected for some network types based on previous findings (e.g., alpha power), the demonstration that this phenomenon holds across all considered network types, and furthermore, can be discerned within individuals, suggests that such network reorganization constitutes a fundamental property of large-scale brain organization. These findings are perhaps even more notable given the marked between-subject variability we observed, to which we turn next.

### Stable Individual Differences of Network Configurations

In addition to the separability of network types based on behavioral state, frequency band, and oscillation metric, our analyses also revealed substantial individual differences discernible above and beyond the commonalities shared across subjects, consistent with other findings in both wakefulness (Chu et al., 2012) and sleep (Cox, Schapiro, Manoach, & Stickgold, 2017; Finelli, Achermann, & Borbély, 2001). Indeed, within-subject network similarity across behavioral states was generally stronger than between-subject similarities for any single state, indicating that idiosyncratic patterns of oscillatory activity tend to persist even during extensive task-induced reorganization. This observation suggests that the same anatomical and functional individual variability measured by MR-based techniques (Bürgel et al., 2006; Finn et al., 2015; Gordon et al., 2015; Mueller et al., 2013) is seen in oscillatory profiles. Furthermore, our finding that individual oscillatory profiles persist across months indicates that these patterns reflect trait rather than state characteristics, similar to our recent findings during sleep (Cox et al., 2017). More generally, these findings are in line with evidence of strong genetic control over brain oscillations (Begleiter & Porjesz, 2006; De Gennaro et al., 2008; Smit, Boomsma, Schnack, Hulshoff Pol, & de Geus, 2012; Smit, Stam, Posthuma, Boomsma, & De Geus, 2008; Van Beijsterveldt & Van Baal, 2002), and with various other MEG/EEG features that exhibit stability across days or even years (Chapeton, Inati, & Zaghloul, 2017; Chu et al., 2012; De Gennaro, Ferrara, Vecchio, Curcio, & Bertini, 2005; Del Pozo-Banos, Alonso, Ticay-Rivas, & Travieso, 2014; Deuker et al., 2009; Hardmeier et al., 2014; Kondacs & Szabó, 1999; Maiorana, La Rocca, & Campisi, 2016; Nikulin & Brismar, 2004; Rocca et al., 2014; Salinsky, Oken, & Morehead, 1991). Given recent links between cognitive functioning and fMRI network structure (Finn et al., 2015; Mueller et al., 2013; Schultz & Cole, 2016) or localized oscillatory activity (Jiang et al., 2015; Klimesch et al., 1990; Park et al., 2014), individual differences in the brain-wide organization of rhythmic activity may similarly map onto behavioral and cognitive differences between individuals.

In further support of the multiplexing hypothesis, classifiers were most successful at identifying individual subjects when information from multiple network types was combined, indicating that these networks each hold unique information. Although task- and rest-based classification rates did not differ dramatically, their combined information led to a further performance increase, suggesting that the same network types offer distinct identifying information during different behavioral states. We also note that not all frequency bands and oscillation metrics contributed equally to classifier success. Alpha activity was sufficient to correctly identify all 14 of our test subjects, although this effect may be due to alpha’s high signal-to-noise ratio, thereby yielding more accurate (and reproducible) oscillatory estimates (Cohen, 2014a). Importantly, however, long-term classification remained high even after exclusion of all alpha-based information, indicating oscillatory fingerprints can be found in multiple frequency bands.

Also noteworthy in this respect is our observation that searchlight-based subject recognition, employing subnetworks centered on each electrode, was highest in frontocentral regions. This finding is reminiscent of recent fMRI evidence showing greatest individual variability of network structure in fronto-parietal areas (Finn et al., 2015; Peña-Gómez, Avena-Koenigsberger, Sepulcre, & Sporns, 2017), raising the possibility that these anatomical areas partly underlie the topographical effects seen here.

### Network Differences and Similarities

The presented findings, derived from a single analytic framework, demonstrate a subtle tension between network differences and similarities. A key example concerns the combination of (1) subject-specific patterns within rest and within task, (2) stable subject-specific patterns *across* rest and task, and (3) robust group-level rest-task differences. Stated differently, multivariate patterns may vary systematically along one set of dimensions (i.e., electrodes/connections) with behavioral state, while varying along other dimensions as a function of subject (and yet others as a function of frequency and oscillation metric). These manifold dynamics are entirely compatible, and whether oscillatory organization of different networks is viewed as mostly similar or mostly different depends on one’s perspective.

In this light, it is worth pointing out that our statistical approach did not directly compare absolute similarity values of different network types. For example, our permutation approach indicated that for both power and phase synchrony, the group-level similarity of alpha rest networks was significantly higher than the baseline similarity of rest networks from different frequency bands. However, average similarity among these alpha rest networks was 0.84 for power, but only 0.28 for phase connectivity (Supporting Information Table 4A, Cox et al., 2018). Thus, for this particular example, connectivity profiles are much more differentiated between individuals than are power topographies. Although it was outside the scope of the present report to systematically compare networks in this fashion, inspection of the Supporting Information Tables (Cox et al., 2018) offers some insights into this complementary perspective of network organization.

We also note that the degree of EEG network similarity between cognitive states (range in S_A_: 0.33–0.73 depending on frequency band and oscillation metric) is much lower than the values around 0.90 typically observed with fMRI (Cole, Bassett, Power, Braver, & Petersen, 2014). Thus, networks may express highly similar hemodynamic interaction patterns across cognitive states while simultaneously showing much more differentiated oscillatory profiles. This may afford EEG more sensitivity to detect network differences relative to fMRI, although this claim should be based on direct comparisons between these recording modalities.

### Biometric Applications of EEG Networks

Our long-term classification results suggest interesting biometric applications for EEG network analyses. Although many EEG features have been examined for their biometric potential (Del Pozo-Banos et al., 2014), large-scale oscillatory patterns, including functional connectivity profiles, have only recently received attention for subject identification (Garau, Fraschini, Didaci, & Marcialis, 2016; Maiorana et al., 2016; Rocca et al., 2014). Compared with these reports, our study examined a substantially longer interval between recordings, demonstrating the long-term permanence of these oscillatory patterns. Indeed, we achieved highly accurate recognition rates without the use of neuronavigational tools to ensure similar electrode cap positioning across visits, thus indicating a remarkable degree of robustness with respect to precise electrode placement. However, we do not claim that subject identification based on oscillatory patterns leads to more distinguishable neural fingerprints compared with approaches employing other EEG features, as we did not perform such comparisons. Likewise, it was not our objective to fine-tune network-based classification by using more sophisticated feature selection and supervised learning approaches. Indeed, the fact that high performance was obtained with relatively straightforward methods underscores the robustness of the observed individual differences.

An open question remains concerning the number of unique individuals our approach could conceivably recognize before different subjects’ network structures begin to overlap and reduce classifier performance. In our sample, networks from different individuals showed high baseline similarity, suggesting that networks cannot freely occupy arbitrary positions in multidimensional space. Moreover, power and connectivity values between adjacent electrodes and frequency bands are typically correlated, resulting in substantial levels of baseline similarity between different network types (see Supporting Information Tables, Cox et al., 2018), further limiting the number of potential network configurations that could be observed. Even with these constraints, however, the number of possible network states is immense. In fact, the dimensionality of this space may be arbitrarily increased by estimating network structure for more fine-grained frequency bands, potentially targeting subject-specific frequencies (Haegens, Cousijn, Wallis, Harrison, & Nobre, 2014), by including additional oscillation metrics (e.g., directional connectivity and cross-frequency coupling measures), or by expanding the number of cognitive states sampled. Moreover, our results show that substantial reductions of network size still resulted in quite accurate performance, indicating that sparse montages already capture a large proportion of between-subject network variability. At the same time, these findings suggest the possibility that the full networks are able to identify a significantly greater number of individuals than we tested here.

### Limitations and Concerns

Several general caveats should be made. First, by constructing networks from ∼5 min data segments, our approach assumes relatively stationary network configurations across this time frame. However, oscillatory network profiles have been shown to fluctuate between different states at timescales of several hundred milliseconds (Baker et al., 2014; Betzel et al., 2012). As we did not analyze network dynamics with this degree of temporal granularity, it is an open question whether each of the multiplexed networks identified here similarly shifts rapidly among multiple network configurations, and if so, how the time courses of these nested network dynamics relate to each other.

Second, although we have discussed the observed network differences from the perspective of neuronal oscillations, oscillations may not always have been present in each of the analyzed frequency bands and/or on each electrode. Thus, our analyses likely captured an unknown mixture of true oscillations and arrhythmic noise within each frequency band (He, 2014), raising the possibility that network differences are related to variability in both types of activity. Somewhat related, our chosen frequency bands, although conventional, may not always offer a clean separation of distinct neural phenomena due to individual differences in spectral peak location (Haegens et al., 2014), harmonics, and/or cross-frequency interactions (Aru et al., 2014). But these confounds would likely result in reduced network separability, suggesting that our findings underestimate the true differences in network structure.

Third, extraneous factors, such as idiosyncratic differences in skull shape or residual muscle and eye artifacts, may have contributed to our findings of individual differences and the long-term stability of EEG characteristics. However, muscle activity is severely attenuated by the surface Laplacian transform (Fitzgibbon et al., 2013), and skull thickness contributes minimally to the scalp EEG (Hagemann, Hewig, Walter, & Naumann, 2008), making it unlikely these factors underlie the reported between-subject variability. Moreover, ocular artifacts were virtually absent during eyes-closed rest segments, and remaining eye artifacts were carefully removed using independent component analysis (ICA). Additional analyses indicated that theta amplitude fluctuations related to ocular activity were effectively abolished by the combination of ICA and the Laplacian, whereas fluctuations in the alpha, beta, and gamma bands were not related to ocular activity at all (Supporting Information Results and Supporting Information Figure 2, Cox et al., 2018), suggesting our findings are of primarily neural origin. That said, we cannot fully exclude the possibility that individual differences in the mapping from neural regions to electrodes, due to variability in head shape, cap size, and/or cap positioning, contributed to observed network differences.

Fourth, a potential issue affecting our analyses concerns volume conduction, whereby activity from a single brain source projects to multiple sensors, giving rise to artificially inflated connectivity estimates between nearby electrodes (S. Palva & Palva, 2012). Although we used a surface Laplacian filter to reduce volume conduction (Perrin et al., 1989), and removed contiguous channels from the connectivity matrix, these approaches do not completely eliminate undesired volume conduction effects. We therefore repeated several key analyses by using connectivity metrics minimally affected by volume conduction (Hipp et al., 2012; Vinck et al., 2011). These examinations revealed that although the use of these control metrics tends to reduce network separability somewhat, our results essentially remained the same, indicating that they cannot be attributed to spurious connectivity resulting from volume conduction (Supporting Information Results, Supporting Information Tables 8–12, Cox et al., 2018). This result likely stems from our unit of analysis being the similarity of distributed *patterns* of activity. In such analyses, individual connection strengths are of secondary relevance, analogous to multivariate fMRI approaches operating on correlated voxels (Tambini & Davachi, 2013). We also tested whether connectivity estimates could be driven by variations in power, but found no such relations, either across individuals or across data segments within the same individual (Supporting Information Results, Cox et al., 2018). On the basis of on these control analyses, as well as the empirical separability of power-, phase-, and amplitude-based networks, we conclude that meaningfully distinct network patterns can be and were derived from sensor-level EEG activity.

Finally, it may be argued that our approach lacks the anatomical specificity afforded by source localization approaches, preventing any interpretation of our findings in terms of neural regions. While this is indeed a limitation, we emphasize that the network similarity approach taken here deliberately abstracts away from particular cortical regions or connections in favor of a more global perspective. Thus, our approach would analyze the same high-level metric of network similarity for source-level data, disregarding the contributions of individual anatomical sources. Although source-based network similarity might offer a more accurate description of true network correspondence, the present sensor-level findings were sufficient to discern separate network types. Indeed, we adopted the network similarity approach because of its potentially increased sensitivity to changes in distributed patterns, offering insights into large-scale brain dynamics not afforded by approaches that rely on mass-univariate statistics (Haxby et al., 2014; Kriegeskorte, 2008). That said, there would be value in characterizing the most consistent and most variable network elements between network types, both in sensor space and anatomically resolved. Such analyses, as well as assessments of graph theoretical measures (Bullmore & Sporns, 2009; van den Heuvel & Sporns, 2013), are outside the scope of the present paper.

### Conclusion

The examination of large-scale oscillatory networks allows for the analysis of brain dynamics at a level unavailable to other techniques. Unlike slower imaging techniques like fMRI, it permits viewing of distinct layers of multiplexed network activity occurring simultaneously, but operating at different oscillatory frequencies and employing distinct modes of neural coordination. The ability to separate these multiplexed signals may prove critical for the unraveling of complex cognition and behavioral control. While this study has focused on continuous oscillatory activity during rest and memory encoding, future studies could investigate network similarity across multiple tasks, and/or examine its relation to behavior. In addition, network similarity measures could be applied in an event-related fashion to examine the network dynamics of cognitive processes with high temporal precision. Finally, these techniques may be harnessed to analyze brain dynamics in neurological and psychiatric disorders, where altered network properties have already been identified (De Vico Fallani et al., 2007; Lynall et al., 2010; Sanz-Arigita et al., 2010). In sum, our observations attest to both the human brain’s incredibly complex oscillatory dynamics, and the wealth of spectral, temporal, and spatial information that can be extracted from EEG signals. Examining oscillatory activity from a network similarity perspective will, we believe, contribute useful insights into the principles of human brain organization.

## METHODS

### Participants

Twenty-one healthy volunteers from the Boston area (8 men, 13 women, mean age ± SD: 22.0 ± 3.0 years, range: 18–31) completed the first visit of this study. Of these, fourteen (8 men, 6 women, 22.6 ± 3.2 years, range: 19–31) returned for a follow-up visit several months later (mean: 154 days, range: 109–231). All reported no history of neurological, psychiatric, or sleep disorders. Participants were instructed to refrain from consuming recreational drugs or alcohol in the 48 hr prior to the study, and to not consume more than one caffeinated beverage on the day of the study. Subjects were compensated monetarily for their participation. All subjects provided written informed consent, and this study was approved by the institutional review board of Beth Israel Deaconess Medical Center.

### Protocol

See Figure 1 for an overview of the protocol. The first visit lasted approximately 5.5 hr. Subjects reported to the laboratory at 1 p.m., provided informed consent, and were prepared for EEG monitoring. Seated approximately 60 cm from a 27-inch computer display, they underwent a series of rest, memory encoding, and memory retrieval blocks, lasting from 2 to 3 p.m. We refer to this first series of rest and task activities as Session A (S_A_). Subjects then remained in the lab watching a 2-hr documentary. Then, from approximately 5:30 to 6 p.m., subjects underwent a second series of recordings (S_B_), during which they engaged in several additional blocks of rest activity and performed delayed memory tests for the material encoded during S_A_. After filling out an exit questionnaire, subjects left the lab around 6:30 p.m. Participants returning for the follow-up visit (S_C_) several months later arrived at the lab at variable times (range: 10 a.m. to 6 p.m.). Following a second informed consent procedure and EEG setup, they underwent a series of rest, encoding, retrieval, and control blocks for about 45 min. Subjects carried out an additional 30 min protocol unrelated to the current study. Total duration of the second visit was about 2.5 hr.

We use the term “block” to refer to a demarcated period of time associated with a particular behavioral state (i.e., rest, encoding, retrieval, and control blocks). Details about the organization of behavioral blocks are described in Supporting Information Methods and in Figure 1 (Cox et al., 2018). Of note, whereas EEG was recorded during all blocks, retrieval was of much shorter duration (∼1 min) than the other blocks, and often of poor quality because of the subjects’ constant movements while operating the mouse. Therefore, we did not analyze the retrieval EEG. We adopt the term “data segment” (or just “segment”) to refer to the EEG obtained during a single rest, encoding, or control (but not retrieval) block. Finally, “task” segments refer to data segments from both encoding and control blocks. Note, however, that a control block was only included in S_C_.

All behavioral blocks were presented using custom software written in Java. Instruction screens occurred throughout the protocol. Subjects advanced to the next screen by pressing a keyboard button. During rest segments, subjects were instructed to quietly rest and relax for 5 min with their eyes closed, while remaining awake. An auditory tone at the end of each rest segment indicated subjects could open their eyes again.

For memory encoding, subjects were instructed to memorize the location of pictures on a 6 × 6 grid. During the task, a square grid of 36 grey squares, subtending approximately 5° of visual angle per tile, and 31° in total, was continually present on the screen. Thirty-six pictures were then shown, one at a time on a unique grid tile, for 2,000 ms with an interstimulus interval of 1,000 ms. This procedure was repeated for a total of three presentations of all 36 pictures. Picture-location combinations and presentation order were randomized for each subject. An encoding block lasted 5 min and 20 s. The control task employed in S_C_ used the same basic design as the encoding protocol, except that the same picture was displayed on each tile, resulting in a perceptually similar experience but with no memory demands.

During memory retrieval, each picture was presented to the right side of the grid. The participant then used the mouse to select the tile on which that picture had been presented, at which point the selected tile turned blue for 400 ms. Then, the tile returned to its gray color and the next picture was presented. Subjects did not receive feedback on their performance. Retrieval was self-paced and lasted 1 to 2 min.

### Data Acquisition and Preprocessing

EEG was collected using 62-channel caps with channel positions in accordance with the 10-20 system. Two Ag/AgCl cup electrodes were attached to the mastoid processes, two around the eyes for electrooculography, and one, as a reference, on the forehead. Channel Afz was used as the ground. An AURA-LTM64 amplifier and TWin software were used for data acquisition (Grass Technologies). Impedances were kept below 25 kΩ, and data were sampled at 400 Hz with hardware high-pass and low-pass filters at 0.1 and 133 Hz, respectively.

All subsequent data processing and analyses were performed in Matlab (the Mathworks, Natick, MA) using custom routines in combination with several open source toolboxes, including EEGlab (Delorme & Makeig, 2004) and Fieldtrip (Oostenveld, Fries, Maris, & Schoffelen, 2011). EEG recordings were divided into data segments based on triggers derived from the stimulus software. Following the removal of eye channels, data segments were re-referenced to average mastoids, high-pass filtered at 0.5 Hz and notch filtered around 60 Hz to suppress line noise. Noisy channels were interpolated using a spherical spline algorithm (EEGlab: *pop_interp*) and excessively noisy time fragments were removed, resulting in an average segment length across all 364 segments of all subjects of 291 ± 22 s (typical range: 209–325 s; one outlier of 48 s). ICA (EEGlab: *runica*) was performed and components reflecting eye movements, eye blinks, muscle activity, and other obvious artifacts were removed. Next, we applied a spatial Laplacian filter (Perrin et al., 1989) by using the CSD toolbox (Kayser & Tenke, 2006). The Laplacian reduces the effects of volume conduction by estimating radial current flow, thereby highlighting local aspects of neural processing and allowing for superior estimates of phase coupling and reducing artificial coupling between electrodes (Cohen, 2014b; Tenke & Kayser, 2015). By decorrelating activity levels across the scalp, this approach “sharpens” network profiles, thereby improving chances of uncovering subtle network differences.

### Power and Functional Connectivity

For each spatially filtered data segment (rest and task) and electrode we estimated power spectral density using Welch’s method with 5-s windows and 50% overlap. Power values were dB transformed according to dB power = 10 × log_10_(power) and averaged across frequency bins corresponding to the theta (3–7 Hz), alpha (8–12 Hz), beta (13–30 Hz), and gamma (32–60 Hz) bands. This yielded, for every subject, data segment and frequency band, a vector *V* of length 60 containing each electrode’s power values. Thus, these vectors reflect the network organization of oscillatory power across the scalp. The dB transformation yielded vectors containing approximately normally distributed values, which is an important assumption for their later use in Pearson correlations. Additionally, as a more concise statistic, we defined global power as the average power across all elements (i.e., electrodes) in a vector: global power = $\frac{1}{n}\sum_{c=1}^{n}V_{c}$, where *c* is the channel number (1, 2, …, 60).

For connectivity, we band-pass filtered the segments by using the abovementioned cut-off frequencies (EEGlab: *pop_eegfiltnew*). We selected these values based on the shape of the filters’ frequency responses, ensuring that there was minimal overlap between adjacent pass-bands. Next, we applied the Hilbert transform to each filtered segment and determined the resulting signals’ instantaneous phase and amplitude. We then subdivided the phase angle and amplitude time series into 10 equally sized smaller data fragments and calculated connectivity separately for each fragment. We performed the fragmenting step to reduce the effect of potential outliers on connectivity estimates, and, for phase synchrony, to allow for nonstationary phase differences within each segment. Fragment length varied across subjects and data segments because data segments had different amounts of artifact removed. Although the number of samples affects the signal-to-noise ratio of the resulting connectivity estimates, individual fragments were sufficiently long (at least 20 s, except for one outlier with fragment lengths of 5 s) that this variation was unlikely to have had a significant influence on subsequent analyses.

Amplitude envelope correlations (Bruns et al., 2000), ranging between −1 and 1, were determined using the Spearman correlation and were assessed between each channel pair’s Hilbert-amplitudes, yielding a matrix *M*_*amp*_ of 60 × 60 connectivity values for every fragment. We used a nonparametric correlation metric because amplitude envelopes are generally not normally distributed. Note that because of the monotonic relation between amplitude and power, amplitude envelope correlations and power envelope correlations are identical in our approach. Phase synchrony was determined following the phase locking value approach (Lachaux et al., 1999). We first determined the phase difference for every channel pair (j, k) at each sample. We then assessed phase synchrony for each channel pair as the length of the average phase difference vector across samples, expressed in the complex plane as:

$${PLV}_{jk}=\left|\frac{1}{n}\;\overset{n}{\underset{t=1}{\sum }}e^{i*(\phi_{j}(t)-\phi_{k}(t))}\right|$$

where *i* is the imaginary operator, *φ* indicates phase (in radians), *t* is the sample, and *j* and *k* index the channels. Phase synchrony values ranged from 0 (random phase relations) to 1 (perfect phase consistency). This resulted in another 60 × 60 matrix *M*_*phase*_, with phase synchrony values between every channel pair for each data fragment. For both amplitude correlation and phase synchrony, we then averaged connectivity estimates across the 10 fragments. We selected the upper triangles of the symmetrical *M*_*amp*_ and *M*_*phase*_ matrices to count each connection only once, resulting in a total of 60 × 59/2 = 1,770 connections. (See Supporting Information Methods, Cox et al., 2018, for calculation of orthogonalized amplitude correlations and weighted phase lag index.)

To further limit the effect of spurious coupling on our results, we removed from each connectivity matrix the 192 elements (11%) that reflect pairs of neighboring electrodes (Fieldtrip: *ft_prepare_neighbours* with *neighbourdist* of 0.55). This yielded, for every subject, data segment, and frequency band, and for both phase synchrony and amplitude correlation, a vector *U* of length 1,578 reflecting the degrees of connectivity between all unique nonneighboring channel pairs. As with power, we also defined a global connectivity metric as the average across all entries in a vector: global connectivity = $\frac{1}{n}\sum_{c=1}^{n}U_{c}$, where *c* is the connection number (1, 2, …, 1,578). For topographical plots of connectivity, we averaged the 60 × 60 matrix *M* (with neighboring and identity connections removed) across one of its dimensions (e.g., row-wise) to obtain the average connectivity between each electrode and all other electrodes.

Connectivity values within each vector *U* were generally not normally distributed, with the degree of skewness differing depending on frequency band, connectivity metric, data segment, and subject. To render the data normally distributed for subsequent Pearson correlation analyses, we first added a value of 1 to each connectivity vector entry to ensure all values were positive. We then performed box-cox power transformations to all vectors, in which the exponent used for transformation was automatically determined for each vector to minimize the standard deviation of the transformed vector (Box & Cox, 1964). Finally, we z-scored the resulting vectors to obtain a standardized appearance when visualized in scatter plots. However, z-scoring does not affect subsequent Pearson correlation statistics. Thus, apart from this power transformation, which was only performed for connectivity vectors, oscillatory power and functional connectivity metrics were processed similarly.

We further assessed whether the use of box-cox transformations and the choice of similarity metric could have affected network similarity estimates. To this end, we assessed network similarity between every possible pair of connectivity vectors (4,403,028 in total) by using either transformed vectors and the Pearson correlation (the “canonical” approach: see Network Similarity and Statistics section), or using untransformed vectors in conjunction with the nonparametric Spearman correlation. The resulting sets of network similarity estimates were highly similar, although significantly different due to the large number of samples (both 0.24 ± 0.14; paired *t*(4,403,027) = 1,758, *p* < 10^−100^). Critically, similarity values were extremely highly correlated between both approaches (*R* = 0.996, *p* < 10^−100^), making it unlikely that downstream analyses were affected by our choice of similarity metric or data transformation.

To directly compare power and connectivity vectors, which were of different length (*V*: 60, *U*: 1,578), we also constructed “power connectivity” vectors of equal size as the connectivity vectors. Specifically, we set the weight of each power “connection” to be the average power of the two involved electrodes. Naturally, this manipulation did not add any novel information, as each of the newly computed values in the larger vector was a linear combination of the original power estimates. As a result, this operation did not influence the similarity among power networks (i.e., power-based network similarity values were identical for vector lengths of 60 and 1,578), while enabling direct comparisons between power and connectivity metrics.

### Network Similarity and Statistics

Our basic approach for assessing the similarity of networks involved computing the Pearson correlation coefficient between two vectors (Figure 3). To aggregate network similarities across more than two networks, we used procedures referred to as representational similarity analysis in the modeling and fMRI literature (Kriegeskorte, 2008). Specifically, we calculated the Pearson correlation between each unique pair of networks, resulting in a large matrix of similarity scores. Then, for specific questions of interest, similarity values were averaged across the relevant entries and compared with a suitable baseline. Thus, we could compare networks within, between, and across subjects, behavioral states, frequency bands, test sessions, and oscillation metrics.

In general, we evaluated network similarity statistically on two levels. In “level-1” analyses we determined how much evidence individual subjects showed for a particular phenomenon. Then, these results were summarized across subjects and appropriately tested for significance. In contrast, “level-2” analyses included all subjects’ networks and asked whether there was reliable support for a particular finding at the group level.

For both level-1 and level-2 analyses, we took a data-driven resampling approach to determine whether observed similarity scores across networks, selected based on a factor of interest, were statistically different from what would be expected under the null hypothesis of equal network similarity across levels of this factor. For every comparison of interest we constructed a null distribution by repeatedly selecting as many random networks as there were included in the original observation (allowing selection of networks included in the original observation). At each iteration, similarity scores between each pair of selected networks were averaged, thereby creating a surrogate distribution of similarity values. The number of resampling iterations we used depended on the number of unique random samples available. If the number of possible combinations was over 1,000, a truly random sample was selected for each of 1,000 iterations (i.e., Monte Carlo sampling). When the number of combinations was lower, every unique combination was sampled exactly once (i.e., permutation sampling). Because of the precise mechanics of shuffling, some level-1 analyses could use the same null distribution for every individual, whereas others required a different baseline distribution for each individual, as outlined in detail in Supporting Information Methods (Cox et al., 2018). For every comparison of interest, we defined baseline similarity as the average similarity across permutations (i.e., the center of the null distribution). In cases where individual subjects had separate null distributions, baseline similarity was defined as the average of these individual baselines.

We assessed significance in several ways. First, for some analyses we use a one-sample *t* test to compare observed level-1 similarity scores to the baseline value. This approach tests whether single-subject effects, as a group, are different from the permutation-derived baseline. Second, for both level-1 and level-2 analyses, we used the null distributions to z-score each observation and calculate the associated *p* values. We then applied the false discovery procedure (Benjamini & Hochberg, 1995) to correct for multiple comparisons (i.e., for multiple subjects, frequency bands, oscillation metrics). We used z-based *p* values, rather than the “raw” permutation-based *p* values, because the latter often severely underestimated the size of the effect. Even with 1,000 iterations the lowest obtainable significance value was *p* < 0.001, while z-based *p* values provide a more accurate estimate of the distance between an observation and its null distribution. Importantly, for some level-1 analyses the number of possible permutations was rather low (e.g., 2 task segments × 4 frequency bands = 8 networks; binomial coefficient $\left(\begin{array}{c} 8\\ 2 \end{array}\right)=28$). As a consequence, the resulting null distributions were based on a limited number of samples and were often not Gaussian shaped. In these cases, z-based *p* values did not optimally capture the size of the effect. Moreover, even when observed similarity values were more extreme than the entire null distribution, their raw permutation-based *p* values were limited by the number of permutations. This state of affairs severely affected subsequent multiple comparison correction with the false discovery procedure. Therefore, we additionally present uncorrected, permutation-based *p* values for these cases, to offer a complete picture of the pattern of results. Finally, meaningful level-1 permutations could not be performed for the comparison of task networks based on the three oscillatory metrics (binomial coefficient: $\left(\begin{array}{c} 6\\ 2 \end{array}\right)=15$), and are not reported. A detailed account of the shuffling mechanics for all different comparisons can be found in Supporting Information Methods (Cox et al., 2018).

We note that whereas we consistently used the false discovery procedure to control the rate of false positives, we did so separately for subsets of analyses (as opposed to across larger sets of analyses or even all possible analyses). For example, for analyses of within-subject network similarity in S_A_, we corrected for 21 comparisons, one for each subject, separately for each network type, rather than correcting for 28 (4 frequencies × 3 metrics × 2 behavioral states) × 21 = 588 comparisons. Besides that we believe the need to control false positives should be balanced against the risk of false negatives, we emphasize that we confirmed our effects in independent sessions S_B_ and S_C_ where possible. Thus, we believe that the nested approach of permutation testing, false discovery rate correction, and cross-session replication adequately corrects for the large number of multiple comparisons we performed.

As an additional tool, we employed multidimensional scaling, as implemented in Matlab’s *mdscale* function, to visualize the similarity of networks. Multidimensional scaling techniques project high-dimensional data points onto a space of lower dimension while optimally preserving the distances between points (Hout, Papesh, & Goldinger, 2013). In our case, each network can be viewed as a point in 60-dimensional (for power topographies) or 1,578-dimensional space (for connectivity patterns), and the distance between networks can be expressed as (1 − Pearson correlation), such that more similar networks are closer together. By projecting these points onto two dimensions, relations between networks of different subjects and types that are not apparent from inspecting the full-dimensional data can be approximately visualized. However, all statistics were performed on the full-dimensional data.

### Classifiers

We used k-nearest neighbor classifiers (Cover & Hart, 1967) as a supervised learning strategy to distinguish between behavioral states within the same session, and to classify subject identity across sessions. In both instances, the algorithm (implemented in Matlab as *fitcknn*) was trained using the correlation distance (1 − Pearson correlation) between each pair of multivariate networks, similar to how we assessed network similarity. Different classifiers were trained for different network types. In the test phase, unseen networks were assigned labels (e.g., “rest” or “task,” or a subject ID) according to the labels in the training set, such that training networks closest to each test network (i.e., more similar networks) contributed more to the final assigned label. This was implemented using an inverse distance-weighting scheme. The number of nearest training networks allowed to vote (i.e., *k*) was set to 5, except for the set of analyses where we investigated classifier performance as a function of *k*.

To classify behavioral states within the same session (i.e., rest vs. task), we used a cross-validated approach in which we repeatedly trained each classifier on the data of all but one subject, and then tested the classifier on the remaining subject’s networks. For cross-session subject recognition, we trained classifiers on data from S_A_ and S_B_ and tested them on data from S_C_. Classifier performance was calculated as the proportion of test networks that were assigned the correct label.

To ascertain whether information pooled across network types improves classification rates of data segments, we combined the information contained by different classifiers. In particular, every individual classifier C_s_ (where *s* is 1, 2, …, 12 for all network types: 4 frequency bands × 3 metrics) returns the posterior probabilities that test network X_i_ belongs to training class Y_j_, where j = 1, 2 for behavioral classifiers (rest or task), and j = 1, 2, …, 21 for subject identity classifiers. For every test network X_i_, we averaged the probabilities across classifiers of interest to obtain “class weights” indicating how likely it is network X_i_ belongs to class Y_j_ (note that resulting values do not necessarily sum to 1 and therefore do not reflect true probabilities). The test network X_i_ was then assigned the class label of the class with the highest class weight, similar to the method used for individual classifiers.

For subject identification, we further averaged these class weights across the multiple data segments derived from each individual subject. In a final merger step, we combined rest and task networks by taking, for each training class Y_j_, the maximum class weight from the composite rest and composite task classifiers, and assigned identity based on this maximum class weight. Classifier performance was evaluated using both binomial tests and permutation tests in which we repeatedly shuffled training labels. For various control analyses, we systematically left out network information from particular frequency bands, oscillation metrics, or data segments prior to merging them.

For searchlight analysis we used the Fieldtrip function *ft_prepare_neighbours* with a neighbor distance of 0.65 to determine the neighborhood structure around each electrode. We then iterated across all electrodes, at each iteration selecting the current electrode, its neighbors, and all nonneighboring connections among them, and trained and tested classifiers on these local networks.

### Data Availability

Power and functional connectivity estimates of all networks are freely available in Matlab format from 10.6084/m9.figshare.5755377. Raw data is available upon request from the lead author.

## ACKNOWLEDGMENTS

We thank Alexandra Morgan for technical assistance, and Mike X. Cohen and Michael Murphy for valuable comments on earlier versions of this manuscript.

## AUTHOR CONTRIBUTIONS

Roy Cox: Conceptualization; Data curation; Formal analysis; Funding acquisition; Investigation; Methodology; Project administration; Software; Visualization; Writing, original draft; Writing, review & editing. Anna C. Schapiro: Conceptualization; Methodology; Writing, review and editing. Robert Stickgold: Conceptualization; Funding acquisition; Methodology; Resources; Supervision; Writing, review and editing.

## FUNDING INFORMATION

This work was supported by grants from the Netherlands Organization for Scientific Research (NWO) to R.C. (446-14-009); the National Institutes of Health to A.C.S. (F32-NS093901), and R.S. (MH048832; MH092638); the Harvard Clinical and Translational Science Center (TR001102); and the Stanley Center for Psychiatric Research at Broad Institute.

## TECHNICAL TERMS

Functional connectivity: Statistical association between time-varying signals measured at distinct sites (e.g., electrodes or voxels), suggestive of direct or indirect communication between the pertaining neural regions.
Connectome: Comprehensive map of connections among brain regions, based on anatomical or functional connectivity.
Cross-frequency coupling: Statistical association between time-varying activity in different frequency bands, suggestive of information transfer between neural processes operating at different timescales.
Multivariate: Relating to the simultaneous analysis of more than one outcome variable.
Surface Laplacian: Spatial filtering approach that, in the context of EEG, accentuates local and minimizes global activity, thereby counteracting volume conduction.
Volume conduction: Phenomenon whereby activity from a single brain source projects to multiple sensors, potentially resulting in artificially inflated functional connectivity.
Multidimensional scaling: Technique to project high-dimensional (or multivariate) data points onto a lower dimensional space while optimally preserving the distances between points.
Supervised learning: Machine learning task of inferring a mathematical mapping from (high-dimensional) data points to their corresponding class labels, (ideally) allowing correct assignment of class labels for unseen test data.
*K*-nearest neighbors classification: Supervised learning algorithm in which each unseen test point is labeled based on the *k* training points it is closest to, according to an arbitrary distance function (e.g., correlation or Euclidean distance).
Biometrics: Metrics of human traits that can be used to label or identify individuals, with applications in digital authentication and access control.
Neuronavigation: Method for registering electrode positions in relation to head shape and/or MRI-based anatomy.

## References

[bib1] Ainsworth M., Lee S., Cunningham M. O., Traub R. D., Kopell N. J., & Whittington M. A. (2012). Rates and rhythms: A synergistic view of frequency and temporal coding in neuronal networks. Neuron, 75(4), 572–583. 10.1016/j.neuron.2012.08.004 22920250

[bib2] Akam T., & Kullmann D. M. (2014). Oscillatory multiplexing of population codes for selective communication in the mammalian brain. Nature Reviews Neuroscience, 15(2), 111–122. 10.1038/nrn3668 24434912 PMC4724886

[bib3] Arnulfo G., Hirvonen J., Nobili L., Palva S., & Palva J. M. (2015). Phase and amplitude correlations in resting-state activity in human stereotactical EEG recordings. NeuroImage, 112, 114–127. 10.1016/j.neuroimage.2015.02.031 25721426

[bib4] Aru J., Aru J., Priesemann V., Wibral M., Lana L., Pipa G., … Vicente R. (2014). Untangling cross-frequency coupling in neuroscience. Current Opinion in Neurobiology, 31, 51–61. 10.1016/j.conb.2014.08.002 25212583

[bib5] Baker A. P., Brookes M. J., Rezek I. A., Smith S. M., Behrens T., Smith P. J. P., & Woolrich M. (2014). Fast transient networks in spontaneous human brain activity. eLife, 2014(3), 1–18. 10.7554/eLife.01867 PMC3965210 24668169

[bib6] Bassett D. S., Meyer-Lindenberg A., Achard S., Duke T., & Bullmore E. (2006). Adaptive reconfiguration of fractal small-world human brain functional networks. Proceedings of the National Academy of Sciences of the United States of America, 103(51), 19518–19523. 17159150 10.1073/pnas.0606005103 PMC1838565

[bib7] Bastos A. M., & Schoffelen J.-M. (2016). A tutorial review of functional connectivity analysis methods and their interpretational pitfalls. Frontiers in Systems Neuroscience, 9(January), 1–23. 10.3389/fnsys.2015.00175 PMC4705224 26778976

[bib8] Begleiter H., & Porjesz B. (2006). Genetics of human brain oscillations. International Journal of Psychophysiology, 60(2), 162–171. 10.1016/j.ijpsycho.2005.12.013 16540194

[bib9] Benjamini Y., & Hochberg Y. (1995). Controlling the false discovery rate: a practical and powerful approach to multiple testing. Journal of the Royal Statistical Society. 10.2307/2346101

[bib10] Betzel R. F., Erickson M. A., Abell M., O’Donnell B. F., Hetrick W. P., & Sporns O. (2012). Synchronization dynamics and evidence for a repertoire of network states in resting EEG. Frontiers in Computational Neuroscience, 6(September), 1–13. 10.3389/fncom.2012.00074 23060785 PMC3460532

[bib11] Box G. E. P., & Cox D. R. (1964). An analysis of transformations. Journal of the Royal Statistical Society. Series B (Methodological), 211–252. 10.2307/2287791

[bib12] Brookes M. J., Liddle E. B., Hale J. R., Woolrich M. W., Luckhoo H., Liddle P. F., & Morris P. G. (2012). Task induced modulation of neural oscillations in electrophysiological brain networks. NeuroImage, 63(4), 1918–1930. 10.1016/j.neuroimage.2012.08.012 22906787

[bib13] Brookes M. J., O’Neill G. C., Hall E. L., Woolrich M. W., Baker A., Palazzo Corner S., … Barnes G. R. (2014). Measuring temporal, spectral and spatial changes in electrophysiological brain network connectivity. NeuroImage, 91, 282–299. 10.1016/j.neuroimage.2013.12.066 24418505

[bib14] Bruns A., Eckhorn R., Jokeit H., & Ebner A. (2000). Amplitude envelope correlation detects coupling among incoherent brain signals. Neuroreport, 11(7), 1509–1514. 10.1097/00001756-200005150-00028 10841367

[bib15] Bullmore E., & Sporns O. (2009). Complex brain networks: graph theoretical analysis of structural and functional systems. Nature Reviews Neuroscience, 10(3), 186–198. 10.1038/nrn2575 19190637

[bib16] Bürgel U., Amunts K., Hoemke L., Mohlberg H., Gilsbach J. M., & Zilles K. (2006). White matter fiber tracts of the human brain: Three-dimensional mapping at microscopic resolution, topography and intersubject variability. NeuroImage, 29(4), 1092–1105. 10.1016/j.neuroimage.2005.08.040 16236527

[bib17] Canolty R. T., Ganguly K., Kennerley S. W., Cadieu C. F., Koepsell K., Wallis J. D., & Carmena J. M. (2010). Oscillatory phase coupling coordinates anatomically dispersed functional cell assemblies. Proceedings of the National Academy of Sciences of the United States of America, 107(40), 17356–17361. 10.1073/pnas.1008306107 20855620 PMC2951408

[bib18] Chapeton J. I., Inati S. K., & Zaghloul K. A. (2017). Stable functional networks exhibit consistent timing in the human brain. Brain, 140(3), 628–640. 10.1093/brain/aww337 28364547 PMC5837656

[bib19] Chu C. J., Kramer M. A., Pathmanathan J., Bianchi M. T., Westover M. B., Wizon L., & Cash S. S. (2012). Emergence of stable functional networks in long-term human electroencephalography. Journal of Neuroscience, 32(8), 2703–2713. 10.1523/JNEUROSCI.5669-11.2012 22357854 PMC3361717

[bib20] Cohen M. X. (2014a). Analyzing neural time series data: Theory and practice. Cambridge, MA: MIT Press.

[bib21] Cohen M. X. (2014b). Comparison of different spatial transformations applied to EEG data: A case study of error processing. International Journal of Psychophysiology, 97(3), 245–257. 10.1016/j.ijpsycho.2014.09.013 25455427

[bib22] Cole M. W., Bassett D. S., Power J. D., Braver T. S., & Petersen S. E. (2014). Intrinsic and task-evoked network architectures of the human brain. Neuron, 83(1), 238–251. 10.1016/j.neuron.2014.05.014 24991964 PMC4082806

[bib23] Congedo M., John R. E., De Ridder D., & Prichep L. (2010). Group independent component analysis of resting state EEG in large normative samples. International Journal of Psychophysiology, 78(2), 89–99. 10.1016/j.ijpsycho.2010.06.003 20598764

[bib24] Cover T., & Hart P. (1967). Nearest neighbor pattern classification. IEEE Transactions on Information Theory, 13(1), 21–27. 10.1109/TIT.1967.1053964

[bib25] Cox R., Schapiro A. C., Manoach D. S., & Stickgold R. (2017). Individual differences in frequency and topography of slow and fast sleep spindles. Frontiers in Human Neuroscience, 11 10.3389/fnhum.2017.00433 PMC5591792 28928647

[bib26] Cox R., Schapiro A. C., & Stickgold R. (2018). Supporting Information for “Variability and stability of large-scale cortical oscillation patterns.” Network Neuroscience, 2(4), 481–512. 10.1162/netn_a_00046 30320295 PMC6175693

[bib27] De Gennaro L., Ferrara M., Vecchio F., Curcio G., & Bertini M. (2005). An electroencephalographic fingerprint of human sleep. NeuroImage, 26(1), 114–122. 10.1016/j.neuroimage.2005.01.020 15862211

[bib28] De Gennaro L., Marzano C., Fratello F., Moroni F., Pellicciari M. C., Ferlazzo F., … Rossini P. M. (2008). The electroencephalographic fingerprint of sleep is genetically determined: A twin study. Annals of Neurology, 64(4), 455–460. 10.1002/ana.21434 18688819

[bib29] De Vico Fallani F., Astolfi L., Cincotti F., Mattia D., Marciani M. G., Salinari S., … Babiloni F. (2007). Cortical functional connectivity networks in normal and spinal cord injured patients: Evaluation by graph analysis. Human Brain Mapping, 28(12), 1334–1346. 10.1002/hbm.20353 17315225 PMC6871447

[bib30] Del Pozo-Banos M., Alonso J. B., Ticay-Rivas J. R., & Travieso C. M. (2014). Electroencephalogram subject identification: A review. Expert Systems with Applications, 41(15), 6537–6554. 10.1016/j.eswa.2014.05.013

[bib31] Delorme A., & Makeig S. (2004). EEGLAB: An open source toolbox for analysis of single-trial EEG dynamics including independent component analysis. Journal of Neuroscience Methods, 134(1), 9–21. 10.1016/j.jneumeth.2003.10.009 15102499

[bib32] Deuker L., Bullmore E. T., Smith M., Christensen S., Nathan P. J., Rockstroh B., & Bassett D. S. (2009). Reproducibility of graph metrics of human brain functional networks. NeuroImage, 47(4), 1460–1468. 10.1016/j.neuroimage.2009.05.035 19463959

[bib33] Fell J., & Axmacher N. (2011). The role of phase synchronization in memory processes. Nature Reviews Neuroscience, 12(2), 105–118. 10.1038/nrn2979 21248789

[bib34] Finelli L. A., Achermann P., & Borbély A. A. (2001). Individual “fingerprints” in human sleep EEG topography.Neuropsychopharmacology, 25(5 Suppl), S57–S62. 10.1016/S0893-133X(01)00320-7 11682275

[bib35] Finn E. S., Shen X., Scheinost D., Rosenberg M. D., Huang J., Chun M. M., … Constable R. T. (2015). Functional connectome fingerprinting: Identifying individuals using patterns of brain connectivity. Nature Neuroscience, 18(11), 1664–1671. 10.1038/nn.4135 26457551 PMC5008686

[bib36] Fitzgibbon S. P., Lewis T. W., Powers D. M. W., Whitham E. W., Willoughby J. O., & Pope K. J. (2013). Surface laplacian of central scalp electrical signals is insensitive to muscle contamination. IEEE Transactions on Biomedical Engineering, 60(1), 4–9. 10.1109/TBME.2012.2195662 22542648

[bib37] Fries P. (2005). A mechanism for cognitive dynamics: Neuronal communication through neuronal coherence. Trends in Cognitive Sciences, 9(10), 474–480. 16150631 10.1016/j.tics.2005.08.011

[bib38] Garau M., Fraschini M., Didaci L., & Marcialis G. L. (2016). Experimental results on multi-modal fusion of EEG-based personal verification algorithms. 2016 International Conference on Biometrics, ICB 2016. 10.1109/ICB.2016.7550080

[bib39] Gordon E. M., Laumann T. O., Adeyemo B., & Petersen S. E. (2015). Individual variability of the system-level organization of the human brain. Cerebral Cortex, (October), bhv239 10.1093/cercor/bhv239 PMC5939195 26464473

[bib40] Gross J., Hoogenboom N., Thut G., Schyns P., Panzeri S., Belin P., & Garrod S. (2013). Speech rhythms and multiplexed oscillatory sensory coding in the human brain. PLoS Biology, 11(12). 10.1371/journal.pbio.1001752 PMC3876971 24391472

[bib41] Haegens S., Cousijn H., Wallis G., Harrison P. J., & Nobre A. C. (2014). Inter- and intra-individual variability in alpha peak frequency. NeuroImage, 92, 46–55. 10.1016/j.neuroimage.2014.01.049 24508648 PMC4013551

[bib42] Hagemann D., Hewig J., Walter C., & Naumann E. (2008). Skull thickness and magnitude of EEG alpha activity. Clinical Neurophysiology, 119(6), 1271–1280. 10.1016/j.clinph.2008.02.010 18387340

[bib43] Hardmeier M., Hatz F., Bousleiman H., Schindler C., Stam C. J., & Fuhr P. (2014). Reproducibility of functional connectivity and graph measures based on the phase lag index (PLI) and weighted phase lag index (wPLI) derived from high resolution EEG. PloS One, 9(10), e108648 10.1371/journal.pone.0108648 25286380 PMC4186758

[bib44] Haxby J. V., Connolly A. C., & Guntupalli J. S. (2014). Decoding neural representational spaces using multivariate pattern analysis. Annual Review of Neuroscience, 37(1), 435–456. 10.1146/annurev-neuro-062012-170325 25002277

[bib45] He B. J. (2014). Scale-free brain activity: Past, present, and future. Trends in Cognitive Sciences, 18(9), 480–487. 10.1016/j.tics.2014.04.003 24788139 PMC4149861

[bib46] Hillebrand A., Barnes G. R., Bosboom J. L., Berendse H. W., & Stam C. J. (2012). Frequency-dependent functional connectivity within resting-state networks: An atlas-based MEG beamformer solution. NeuroImage, 59(4), 3909–3921. 10.1016/j.neuroimage.2011.11.005 22122866 PMC3382730

[bib47] Hipp J. F., Hawellek D. J., Corbetta M., Siegel M., & Engel A. K. (2012). Large-scale cortical correlation structure of spontaneous oscillatory activity. Nature Neuroscience, 15(6), 884–890. 10.1038/nn.3101 22561454 PMC3861400

[bib48] Hipp J. F., & Siegel M. (2015). BOLD fMRI correlation reflects frequency-specific neuronal correlation. Current Biology, 25(10), 1368–1374. 10.1016/j.cub.2015.03.049 25936551

[bib49] Honkanen R., Rouhinen S., Wang S. H., Palva J. M., & Palva S. (2015). Gamma oscillations underlie the maintenance of feature-specific information and the contents of visual working memory. Cerebral Cortex, 25(10), 3788–3801. 10.1093/cercor/bhu263 25405942

[bib50] Hout M. C., Papesh M. H., & Goldinger S. D. (2013). Multidimensional scaling. Wiley Interdisciplinary Reviews: Cognitive Science, 4(1), 93–103. doi: 10.1002/wcs.1203 23359318 PMC3555222

[bib51] Jiang H., van Gerven M. A. J., & Jensen O. (2015). Modality-specific alpha modulations facilitate long-term memory encoding in the presence of distracters. Journal of Cognitive Neuroscience, 27(3), 583–592. 10.1162/jocn_a_00726 25244116

[bib52] Kayser J., & Tenke C. E. (2006). Principal components analysis of Laplacian waveforms as a generic method for identifying ERP generator patterns: I. Evaluation with auditory oddball tasks. Clinical Neurophysiology, 117(2), 348–368. 10.1016/j.clinph.2005.08.034 16356767

[bib53] Keitel A., & Gross J. (2016). Individual human brain areas can be identified from their characteristic spectral activation fingerprints. PLOS Biology, 14(6), e1002498 10.1371/journal.pbio.1002498 27355236 PMC4927181

[bib54] Klimesch W., Schimke H., Ladurner G., & Pfurtscheller G. (1990). Alpha frequency and memory performance. Journal of Psycho physiology, 4(4), 381–390.

[bib55] Kondacs A., & Szabó M. (1999). Long-term intra-individual variability of the background EEG in normals. Clinical Neurophysiology, 110(10), 1708–1716. 10.1016/S1388-24579900122-4 10574286

[bib56] Kriegeskorte N. (2008). Representational similarity analysis—Connecting the branches of systems neuroscience. Frontiers in Systems Neuroscience, 2(November), 4 10.3389/neuro.06.004.2008 19104670 PMC2605405

[bib57] Lachaux J.-P., Rodriguez E., Martinerie J., & Varela F. J. (1999). Measuring phase synchrony in brain signals. Human Brain Mapping, 8(4), 194–208. 10.1002/(SICI)1097-0193(1999)8:4<194::AID-HBM4>3.0.CO;2-C 10619414 PMC6873296

[bib58] Lopes da Silva F. (2013). EEG and MEG: Relevance to neuroscience. Neuron, 80(5), 1112–1128. 10.1016/j.neuron.2013.10.017 24314724

[bib59] Lynall M. E., Bassett D. S., Kerwin R., McKenna P. J., Kitzbichler M., Muller U., & Bullmore E. (2010). Functional connectivity and brain networks in schizophrenia. Journal of Neuroscience, 30(28), 9477–9487. 10.1523/JNEUROSCI.0333-10.2010 20631176 PMC2914251

[bib60] Maiorana E., La Rocca D., & Campisi P. (2016). On the permanence of EEG signals for biometric recognition. IEEE Transactions on Information Forensics and Security, 11(1), 163–175. 10.1109/TIFS.2015.2481870

[bib61] Mueller S., Wang D., Fox M. D., Yeo B. T. T., Sepulcre J., Sabuncu M. R., … Liu H. (2013). Individual variability in functional connectivity architecture of the human brain. Neuron, 77(3), 586–595. doi: 10.1016/j.neuron.2012.12.028 23395382 PMC3746075

[bib62] Nikulin V. V., & Brismar T. (2004). Long-range temporal correlations in alpha and beta oscillations: Effect of arousal level and test-retest reliability. Clinical Neurophysiology, 115(8), 1896–1908. 10.1016/j.clinph.2004.03.019 15261868

[bib63] Oostenveld R., Fries P., Maris E., & Schoffelen J. M. (2011). Fieldtrip: Open source software for advanced analysis of MEG, EEG, and invasive electrophysiological data. Computational Intelligence and Neuroscience, 2011 . 10.1155/2011/156869 PMC3021840 21253357

[bib64] Palva J. M., Monto S., Kulashekhar S., & Palva S. (2010). Neuronal synchrony reveals working memory networks and predicts individual memory capacity. Proceedings of the National Academy of Sciences of the United States of America, 107(16), 7580–7585. 10.1073/pnas.0913113107 20368447 PMC2867688

[bib65] Palva S., & Palva J. M. (2012). Discovering oscillatory interaction networks with M/EEG: Challenges and breakthroughs. Trends in Cognitive Sciences, 16(4), 219–229. 10.1016/j.tics.2012.02.004 22440830

[bib66] Panzeri S., Macke J. H., Gross J., & Kayser C. (2015). Neural population coding: Combining insights from microscopic and mass signals. Trends in Cognitive Sciences, 19(3), 162–172. 10.1016/j.tics.2015.01.002 25670005 PMC4379382

[bib67] Park H., Lee D. S., Kang E., Kang H., Hahm J., Kim J. S., … Jensen O. (2014). Blocking of irrelevant memories by posterior alpha activity boosts memory encoding. Human Brain Mapping, 35(8), 3972–3987. 10.1002/hbm.22452 24522937 PMC6869719

[bib68] Peña-Gómez C., Avena-Koenigsberger A., Sepulcre J., & Sporns O. (2017). Spatiotemporal network markers of individual variability in the human functional connectome. Cerebral Cortex, (2014), 1–13. 10.1093/cercor/bhx170 28981611 PMC6041986

[bib69] Perrin F., Pernier J., Bertrand O., & Echallier J. F. (1989). Spherical splines for scalp potential and current density mapping. Electroencephalography and Clinical Neurophysiology, 72(2), 184–187. 2464490 10.1016/0013-4694(89)90180-6

[bib70] Pfurtscheller G. (1992). Event-related synchronization (ERS): An electrophysiological correlate of cortical areas at rest. Electroencephalography and Clinical Neurophysiology, 83(1), 62–69. 10.1016/0013-4694(92)90133-3 1376667

[bib71] Rocca D. La, Campisi P., Vegso B., Cserti P., Kozmann G., Babiloni F., & Fallani F. D. V. (2014). Human brain distinctiveness based on EEG spectral coherence connectivity. IEEE Transactions on Biomedical Engineering, 61(9), 2406–2412. 10.1109/TBME.2014.2317881 24759981

[bib72] Salinsky M. C., Oken B. S., & Morehead L. (1991). Test-retest reliability in EEG frequency analysis. Electroencephalography and Clinical Neurophysiology, 79(5), 382–392. 10.1016/0013-4694(91)90203-G 1718711

[bib73] Sanz-Arigita E. J., Schoonheim M. M., Damoiseaux J. S., Rombouts S. A. R. B., Maris E., Barkhof F., … Stam C. J. (2010). Loss of “small-world” networks in Alzheimer’s disease: Graph analysis of fMRI resting-state functional connectivity. PLoS ONE, 5(11). 10.1371/journal.pone.0013788 PMC2967467 21072180

[bib74] Schultz D. H., & Cole M. W. (2016). Higher intelligence is associated with less task-related brain network reconfiguration. Journal of Neuroscience, 36(33), 8551–8561. 10.1523/JNEUROSCI.0358-16.2016 27535904 PMC4987432

[bib75] Schyns P. G., Thut G., & Gross J. (2011). Cracking the code of oscillatory activity. PLoS Biology, 9(5), e1001064 10.1371/journal.pbio.1001064 21610856 PMC3096604

[bib76] Siegel M., Donner T. H., & Engel A. K. (2012). Spectral fingerprints of large-scale neuronal interactions. Nature Reviews Neuro science, 13(February), 20–25. 10.1038/nrn3137 22233726

[bib77] Siems M., Pape A.-A., Hipp J. F., & Siegel M. (2016). Measuring the cortical correlation structure of spontaneous oscillatory activity with EEG and MEG. NeuroImage, 129, 345–355. 10.1016/j.neuroimage.2016.01.055 26827813

[bib78] Smit D. J. A., Boomsma D. I., Schnack H. G., Hulshoff Pol H. E., & de Geus E. J. C. (2012). Individual differences in EEG spectral power reflect genetic variance in gray and white matter volumes. Twin Research and Human Genetics, 15(3), 384–392. 10.1017/thg.2012.6 22856372

[bib79] Smit D. J. A., Stam C. J., Posthuma D., Boomsma D. I., & De Geus E. J. C. (2008). Heritability of “small-world” networks in the brain: A graph theoretical analysis of resting-state EEG functional connectivity. Human Brain Mapping, 29(12), 1368–1378. 10.1002/hbm.20468 18064590 PMC6870849

[bib80] Tambini A., & Davachi L. (2013). Persistence of hippocampal multivoxel patterns into postencoding rest is related to memory. Proceedings of the National Academy of Sciences of the United States of America, 110(48), 19591–19596. 10.1073/pnas.1308499110 24218550 PMC3845130

[bib81] Tenke C. E., & Kayser J. (2015). Surface Laplacians (SL) and phase properties of EEG rhythms: Simulated generators in a volume-conduction model. International Journal of Psychophysiology, 97(3), 285–298. 10.1016/j.ijpsycho.2015.05.008 26004020 PMC4537832

[bib82] Thut G., Miniussi C., & Gross J. (2012). The functional importance of rhythmic activity in the brain. Current Biology, 22(16), R658–R663. 10.1016/j.cub.2012.06.061 22917517

[bib83] Van Beijsterveldt C. E. M., & Van Baal G. C. M. (2002). Twin and family studies of the human electroencephalogram: A review and a meta-analysis. Biological Psychology, 61(1–2), 111–138. 10.1016/S0301-0511(02)00055-8 12385672

[bib84] van den Heuvel M. P., & Sporns O. (2013). Network hubs in the human brain. Trends in Cognitive Sciences, 17(12), 683–696. 10.1016/j.tics.2013.09.012 24231140

[bib85] Vinck M., Oostenveld R., Van Wingerden M., Battaglia F., & Pennartz C. M. A. (2011). An improved index of phase-synchronization for electrophysiological data in the presence of volume-conduction, noise and sample-size bias. NeuroImage, 55(4), 1548–1565. 10.1016/j.neuroimage.2011.01.055 21276857

[bib86] Watrous A. J., Fell J., Ekstrom A. D., & Axmacher N. (2015). More than spikes: Common oscillatory mechanisms for content specific neural representations during perception and memory. Current Opinion in Neurobiology, 31, 33–39. 10.1016/j.conb.2014.07.024 25129044 PMC4329113

[bib87] Watrous A. J., Tandon N., Conner C. R., Pieters T., & Ekstrom A. D. (2013). Frequency-specific network connectivity increases underlie accurate spatiotemporal memory retrieval. Nature Neuroscience, 16(3), 349–356. 10.1038/nn.3315 23354333 PMC3581758

