# Supplementary material for: Variability and stability of large-scale cortical oscillation patterns
Source: Netw Neurosci. 2018 Oct 1;2(4):481–512. doi: 10.1162/netn_a_00046 (PMC6175693; doi:10.1162/netn_a_00046)
Supplement: Supplementary file 1 [file netn-02-481-s001.pdf]

Cox, R., Schapiro, A. C., Stickgold, R. (2018). Supporting Information for “Variability and stability of large-scale cortical oscillation patterns.” Network Neuroscience, 2(4), 481-512. https://doi.org/10.1162/netn_a_00046

Variability and Stability of Large-Scale Cortical Oscillation Patterns

Roy Cox, Anna C Schapiro, Robert Stickgold

Supplementary Results

Memory performance

During S_A, subjects memorized the locations of pictures in two distinct semantic categories (animals and vehicles) in separate runs, with order counterbalanced, and retrieved them 5 minutes after their respective encoding block (Fig. 1). Retrieval accuracy averaged across the two runs was 52 ± 22% (mean ± SD; chance: 3%), with a significant practice effect (better performance on the second run: 59 ± 23% vs. 46 ± 24%; t(20)=-3.5, P=0.002). Performance on the two runs was highly correlated within subjects (R=0.74, P=0.0001). In a 2x2 mixed ANOVA with between-subject factor ORDER (animals or vehicles first) and within-subject factor RUN (first/second), no significant main effect of ORDER or ORDER*RUN interaction was found (both P>0.4), although there was a significant main effect of RUN (F(1,19)=12.0, P=0.003) corresponding to the practice effect noted above. Performance for the two semantic categories (independent of order) did not differ substantially either (t(20)=0.2, P=0.82), but was again highly correlated within subjects (R=0.62, P=0.003).

Two hours after the end of S_A, in S_B, participants performed delayed retrieval tests for the material memorized in the two encoding blocks of S_A. Absolute performance was reduced compared to immediate tests in S_A for both delayed tests in S_B (run 1: 41 ± 24%; t(20)=2.9, P=0.009; run 2: 55 ± 25%; t(20)=1.9, P=0.08), resulting in retention rates of 85 ± 29% and 89 ± 26% that were similar for both tests (t(20)=-0.7, P=0.51).

At follow-up several months later (S_C), 14 returning subjects memorized and retrieved a third set of visuospatial associations. They also performed a non-learning control task where a single stimulus was repeatedly presented three times at each location. They were told to simply view these presentations and that there would not be any memory test. The order of the learning and control tasks was counterbalanced across subjects and did not affect performance (56.3 ± 23.1% vs. 56.3 ± 29.5 %; t(12)=0, P=1). Paired t-tests for these 14 subjects indicated that S_C performance was similar to average S_A scores (56 ± 25% vs. 50 ± 25%; t(13)=1.5, P=0.15), and that these scores were highly correlated within subjects across this 3-8 month period (R=0.81, P=0.0005). Together, these findings indicate that individual variation in episodic memory on this task is a robust trait.

Global and topographical oscillatory activity

The network similarity approach in the main article compares the *relative* distributions of oscillatory activity across the cortex between pairs of vectors. In order to also obtain an overall picture of *absolute* oscillatory activity, we examined global

spectral power (i.e., averaged across all channels), for rest and task segments during S_A (Supplementary Fig. 1A, top). A 4 (frequency) x 2 (rest/task) repeated-measures ANOVA revealed significant main and interaction effects (all $P<0.04$). In line with typical 1/f frequency scaling, lower frequency bands generally showed greater power than higher bands (paired t-tests: all $P_{corr}<10^{-9}$), except for the theta and alpha pair which, due to prominent alpha peaks, showed the reverse pattern during rest [$t(20)=-6.3$, $P_{corr}<10^{-5}$] and did not differ during task [$t(20)=1.7$, $P_{corr}=0.11$). Comparing rest and task, theta [$t(20)=3.3$, $P_{corr}=0.005$] and especially alpha activity [$t(20)=8.5$, $P_{corr}<10^{-6}$] were distinctly higher during eyes-closed rest periods compared to task segments, while the reverse was true for gamma power ($t(20)=-5.5$, $P_{corr}<10^{-4}$). Accompanying topographical plots demonstrate the regional contributions to these global effects (Supplementary Fig. 1A, bottom).

Similarly, to assess overall levels of absolute functional connectivity, we determined the average connection strength of each connectivity vector (using raw values before normalization) and averaged the resulting values separately for task and resting-state segments. For both amplitude- (Supplementary Fig. 1B, top) and phase- (Supplementary Fig. 1C, top) based connectivity metrics, 4x2 ANOVAs yielded reliable main and interaction effects (all $P<0.01$). Regarding rest-task differences, rest periods exhibited stronger alpha connectivity [amplitude-based: $t(20)=6.8$, $P_{corr}<10^{-5}$; phase-based: $t(20)=4.8$, $P_{corr}=0.0004$] and beta connectivity [amplitude-based: $t(20)=4.7$, $P_{corr}=0.0003$; phase-based: $t(20)=1.9$, $P_{corr}=0.07$]. During task, gamma connectivity was stronger for both metrics [amplitude: $t(20)=-2.3$, $P_{corr}=0.04$; phase: $t(20)=-2.1$, $P_{corr}=0.06$], as was phase-based theta connectivity [$t(20)=4.0$, $P_{corr}=0.002$]. Considering frequencies, all individual frequency pairs demonstrated significant differences for amplitude-based rest connectivity (Supplementary Fig. 1B, top, blue bars) and phase-based task connectivity (Supplementary Fig. 1C, top, red bars). For amplitude-based task segments and phase-based rest segments, 3/6 and 4/6 frequency comparisons were significant (all $P_{corr}<0.05$). Topographical plots visualizing the average connectivity at every electrode site with the rest of the brain indicate the contributions from specific cortical regions to these global effects (bottoms of Supplementary Fig. 1BC).

We also examined global and topographical power and connectivity dynamics during S_C and found very similar results. We note that while some features of global connectivity resemble the power profile (e.g., highest values in alpha band during rest), others do not (e.g., greater theta power during rest but stronger theta phase connectivity during task; rest-task differences in beta connectivity but not beta power). Analogous to global effects, topographical maps for power, amplitude correlation, and phase synchrony suggest both regional similarities across oscillatory metrics (e.g., posterior activity during rest), and marked differences (e.g., central distributions for beta amplitude-based connectivity not seen for power or phase synchrony).

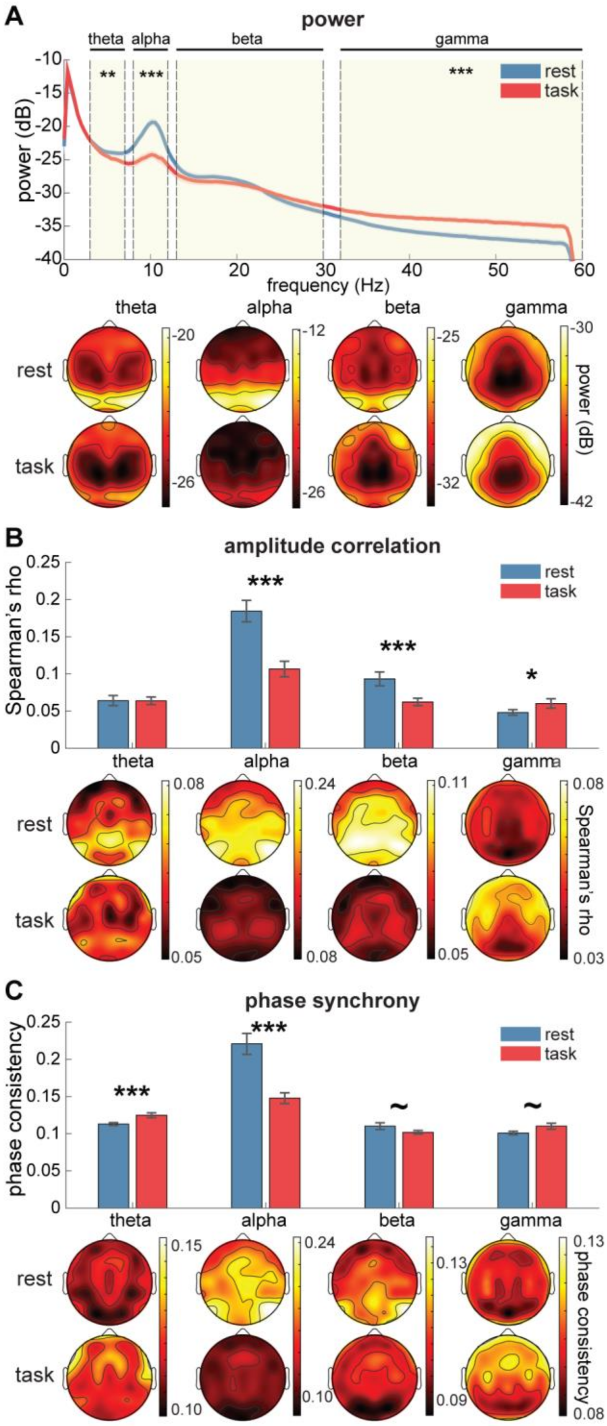

Supplementary Figure 1. Global and local power and connectivity during S_A rest and task segments. (**A**) Top: Power averaged across all electrodes. Rest periods were marked by greater alpha and theta power but reduced gamma activity. Bottom: Topographical distributions of power show typical posterior distributions of alpha activity, with greater power during rest. Global amplitude correlation (**B**), and global phase synchrony (**C**) averaged across all electrode pairs (top), and as topographical plots where each electrode's connectivity is color-coded according to its average connectivity with all other electrodes (bottom). Symbols indicate significantly (~: $P<0.1$; *: $P<0.05$; **: $P<0.01$; ***: $P<0.001$) different global power or connectivity between rest and task. For details regarding statistical differences between frequency bands see Supplementary Results.

Combined, these findings indicate that global and topographical measures of absolute oscillatory activity vary with frequency and are affected differently by task and rest conditions in separate frequency bands. Moreover, different oscillatory metrics appear sensitive to different aspects of brain dynamics. Thus, these analyses support the general notion of separable network types, although they do not allow direct comparisons between network patterns as reported in the main text.

Short-term network similarity

The analyses in the main article indicated high within-subject network similarity within a single recording session (e.g., within S_A). While our classifier results demonstrated the long-term stability of these patterns in terms of recognition performance, these analyses did not directly assess network similarity across time. To this end, we first employed our similarity-and-permutation approach to study the stability of network structure across sessions by comparing networks from S_A and S_B, which were spaced 2 h apart. We compared the observed within-subject, between-

session similarity scores to null distributions in which we paired each subject's networks from one session with networks randomly selected across subjects from the other session. We did this for all network types, and separately for $rest_A$-$rest_B$ and $task_A$-$rest_B$ comparisons.

Both sets of analyses yielded within-subject similarity values that were consistently greater than those obtained by chance (Supplementary Table 6A). At the group-level, greater than chance network stability occurred for every network type, and for both rest-rest, and task-rest comparisons (one-sample t-tests: $P<0.005$ for each comparison). At the single subject-level, 90-100% of individuals reached significance for rest-rest stability, depending on network type, while 50-100% showed significantly stable network organizations between rest and task segments across the 2 h interval. Interestingly, this pattern of results did not deviate substantially from the within-session analyses (Supplementary Table 1A). Across network types, proportions of subjects showing significant same-session and between-session stability were similar for rest segments [96.4 ± 4.1 vs. 97.6 ± 3.2%, $t(11)=-1.4$, $P=0.19$, paired t-test], but decreased over time for rest-task comparisons [86.9 ± 9.3 vs. 72.2 ± 15.0%, $t(11)=6.6$, $P<10^{-4}$]. These findings suggest only a modest change in oscillatory brain patterns over a 2 h period.

Long-term network stability

Next, we analyzed network similarity across the 3–8 month interval from S_{AB} to S_C. Because rest segments from S_A and S_B were highly similar, we treated these data as stemming from one session. We performed four sets of comparisons between S_{AB} and S_C: rest-rest, task-task, rest-task, and task-rest.

At the group-level, above-chance network similarity values were obtained for all network types, both for rest-rest and task-task comparisons (all $P<0.02$). For rest-task comparisons, all comparisons were significant at $P<0.05$, while 10 of the 12 network types reached significance for task-rest analyses. Assessing single-subject-level statistics, we observed sizable proportions of individual subjects with significant similarity scores. Between 43 and 100% of subjects exhibited significant cross-session $S_{AB–C}$ network stability, depending on frequency band, oscillation metric and behavioral states compared (Supplementary Table 6BC).

Interestingly, we found that network stability across network types decreased by only 9% from $S_{A–B}$ to $S_{AB–C}$ for rest-rest networks [subject proportions reaching significance: 97.6 ± 3.2 vs. 88.7 ± 10.3%; $t(11)=3.1$, $P=0.01$], and non-significantly for rest-task networks [72.2 ± 15.0 vs. 70.8 ± 15.1%; $t(11)=0.5$, $P=0.63$]. Thus, there appears to be very little change in individual network structure over months.

Segment classification control analyses

In the main text, we performed long-term classification analyses of individual data segments. However, since we had different numbers of rest and task segments available (both in S_{AB} and S_C), this could have biased our results. In a control analysis, we limited the number of training and test segments available to rest classifiers to two of each (S_A: $rest_{A2}$ and $rest_{A5}$; S_C: $rest_{C2}$ and $rest_{C4}$) to match the number of segments

used for task classifiers (Supplementary Table 6). As expected, this resulted in reduced performance for rest classifiers, but also for rest compared to task classifiers [t(11)=-3.6, P=0.005], although the poorest performing classifier still correctly identified 25% of segments and performed better than all attempts with reshuffled training labels (both binomial and permutation: P<0.001).

Subject identification control analyses

In the main text, we performed long-term classification analyses of individuals by pooling across segments from the same individual. In a set of control analyses, we performed the subject classification procedure using only two training (S_A: $rest_{A2}$ and $rest_{A5}$) and two testing rest segments (S_C: $rest_{C2}$ and $rest_{C4}$) and two task segments (for both training and testing) to match the number of segments available to task classifiers. This resulted in successful identification for 93% of individuals (13 of 14). Next, including all rest and task segments again, we evaluated whether our results might depend on the number of nearest training networks evaluated when labeling a test network. Varying the parameter *k* between 1 and 10 we found that for *k*=1 and *k*=2 one subject was misclassified, but accuracy was stable at 100% for all higher values. We also tested the classifiers trained on S_{AB} rest networks on S_C task segments, and vice versa, and combined their votes. This yielded 79% correct subject identification, demonstrating robust network stability across both time and behavioral state.

No relation between EEG and ocular activity

We performed a set of analyses to assess the influence of ocular activity (eye blinks, saccades) on the EEG signal. To this end, we selected two representative subjects' $task_{A1}$ segments (when eye activity is most profound), and compared the electrooculography (EOG) signal to various traces derived from frontal channel Fpz (where eye artifacts are expected to be particularly strong). As depicted visually in Supplementary Fig. 2 for subject 11, the EOG signal was substantially related to the raw unfiltered EEG trace (subject 11: R=0.34; subject 6: R=0.72) and the envelope of the theta-filtered signal (R=0.16 and R=0.18). These correlations were greatly reduced after ICA cleaning and the Laplacian transformation (Laplacian-unfiltered: R=0.14 and R=0.06; Laplacian-theta: R=0.00 and R=–0.09). Amplitude envelopes in the alpha, beta, and gamma bands showed poor correlations with the EOG channel both before and after the ICA/Laplacian procedure (all |R|<0.09). In sum, these analyses suggest that the data used for assessing network similarity did not reflect eye activity.

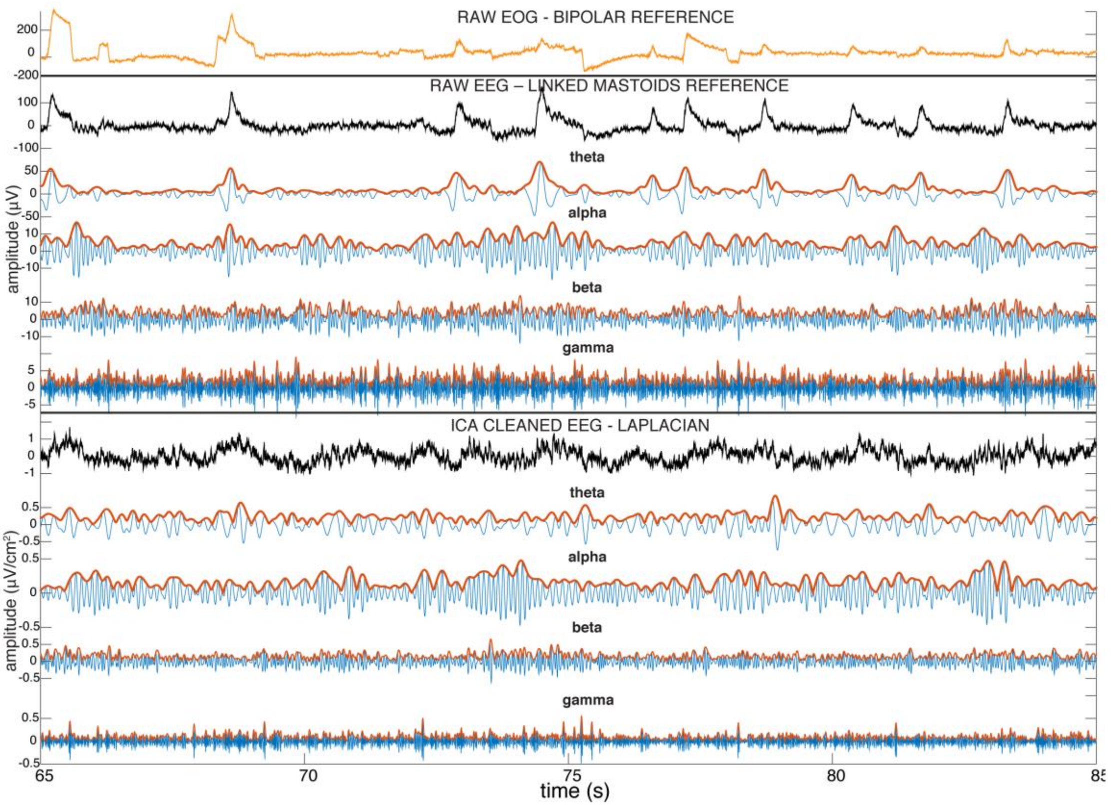

Supplementary Figure 2. Relation between eye channel activity and EEG channel Fpz for subject 11. From top to bottom: EOG (yellow) recorded with bipolar montage of horizontally placed electrodes showing both eye movements and blinks; raw unfiltered EEG (black) showing clear blinking activity; activity filtered in each of the four frequency bands (blue) with corresponding envelope amplitudes (red), showing high sensitivity of the theta band to eye blinks; unfiltered EEG after ICA and Laplacian transformation (black); frequency-specific activity (blue) and corresponding envelopes (red) of Laplacian signal showing no relation to EOG signal.

No relation between power and functional connectivity

The observation that absolute oscillatory activity (i.e., global: power and connectivity averaged across all electrodes/connections; or topographical: power per electrode or average connectivity with every electrode) show different activity patterns for power and connectivity (Supplementary Fig. 1) suggests that functional connectivity was not driven by variations in power. We performed additional analyses to examine whether power and functional connectivity are trivially related. In this scenario, greater power leads to enhanced signal to noise ratios, improved phase and amplitude estimates, and thereby inflated connectivity. Thus, if functional connectivity were due to power fluctuations, power and connectivity should be correlated.

We investigated this relation in two ways. First, we correlated S_A and S_B global absolute power and connectivity values across subjects, separately for each frequency band, the two connectivity metrics, and behavioral state. We combined rest segments from S_A and S_B as we found these to be highly similar (see *Short-term network similarity*). However, we found no evidence for a systematic relation between power

and either phase- or amplitude-based connectivity. Specifically, power was positively related to amplitude-based connectivity, but only in the alpha band during task performance (P_{corr} = 0.03). None of the other 15 relations we examined showed significant correlations (all P_{corr}>0.11). Thus, subjects with greater overall connectivity were not typically the ones with greatest overall power.

Second, we investigated the within-subject relation between power and connectivity by correlating oscillatory estimates across data segments, separately for every subject, frequency band, and connectivity metric. Here, we only used rest segments to prevent a confounding influence of behavioral state, and again combined segments from S_A and S_B to obtain more data points for each correlation (11 in total). Correcting for multiple comparisons separately for each frequency band and connectivity metric, we observed that only three or four of the 21 subjects showed a significant association between power and connectivity across segments in each frequency band. Moreover, different subjects expressed these relations in different spectral bands, and the direction of correlations - positive or negative - was variable across subjects and spectral bands. Thus, we did not find any consistent evidence that data segments with greater power were associated with enhanced connectivity within the same individual.

In sum, neither between- nor within-subject variability in global connectivity appeared to be consistently related to differences in power, and these metrics therefore appear to capture distinct aspects of oscillatory processing. Still, we cannot exclude the possibility that, even after the surface Laplacian transformation and removal of neighboring connections, local power modulations might still affect local connectivity estimates. However, we emphasize that for the network similarity analyses presented in the main article, we are concerned with the organization of large-scale oscillatory patterns, not with the strength of individual connections.

Control connectivity metrics

Our main analyses are based on functional connectivity metrics that are susceptible to undesired volume conduction effects, such that connectivity estimates could be inflated by the same brain source projecting to nearby electrodes. Although we strived to minimize this possibility by applying a surface Laplacian to our data, as well as removing neighboring channels from the connectivity matrix, it is in principle possible that our results are still impacted by residual artifactual connectivity.

To address this issue, we performed control analyses using phase- and amplitude-based connectivity metrics designed to minimize the effects of volume conduction. Specifically, we used the weighted phase lag index (wPLI) (Vinck, Oostenveld, Van Wingerden, Battaglia, & Pennartz, 2011), a measure of phase synchrony that de-weights zero phase (and antiphase) connectivity, and orthogonalized amplitude envelope correlations (Hipp, Hawellek, Corbetta, Siegel, & Engel, 2012), a technique developed to remove trivial co-fluctuations between signal amplitudes at different recording locations due to measuring the same brain sources (see *Supplementary Methods*). We note that while these metrics result in a rigorous removal of volume conduction effects, they are also likely to remove true

physiological connectivity, as electrophysiological recordings in both animals (Roelfsema, Engel, König, & Singer, 1997; Stujenske, Likhtik, Topiwala, & Gordon, 2014) and humans (Slotnick, Moo, Kraut, Lesser, & Hart, 2002) have demonstrated the existence of long-range synchrony with zero time lag, which may emerge naturally from a fundamental wiring motif (Vicente, Gollo, Mirasso, Fischer, & Pipa, 2008). Hence, even if no undesired volume conduction effects were to exist, adoption of these control metrics could still be expected to lead to a reduction in network separability due to the removal of true connectivity.

Absolute connectivity and network similarity

We first compared absolute phase-based connectivity, across all 1,578 included connections, between our standard and control connectivity metrics for a representative subject's $rest_{A1}$ segment. Although we observed significant differences, partialing out zero-phase lag connectivity did not lead to large or consistent changes in connectivity across frequency bands (Supplementary Table 8). Repeating these analyses for amplitude correlations, we found standard amplitude correlations to be substantially and significantly greater than the control approach, consistent with published observations (Hipp et al., 2012).

Following box-cox transformations and z-scoring of each network vector, we next assessed network similarity as the Pearson correlation between network pairs, similar to our approach in the main text. Examining these similarity values between the example subject's $rest_{A1}$ and $rest_{A2}$ segments, we observed network similarity based on standard metrics to be substantially higher than network similarity based on the control metrics, for both phase- and amplitude-based connectivity (Supplementary Table 9). However, these lowered network similarity estimates for the control metrics were still highly significant (all $P<0.001$, except for theta amplitude-based connectivity).

Based on these indications that control metrics of functional connectivity appear capable of gauging the similarity of large-scale oscillatory patterns beyond noise levels, we proceeded to repeat several key analyses reported in the main text. Focusing on S_A, we compared network similarity within and between subjects, behavioral states, and frequency bands, following the same statistical permutation procedures as employed for our main analyses. In addition, we assessed long-term subject classification of data segments from S_{AB} to S_C.

Network consistency within individuals

First, we assessed network consistency within individuals for S_A networks based on phase synchrony. We employed the same permutation approach with correction for multiple comparisons (one for each subject) as used for the findings in the main text. We observed that for both rest and task, as well as *across* rest and task, networks based on wPLI were more similar within than between subjects to a similar degree as networks based on standard phase synchrony (Supplementary Table 10). Assessing whether subject proportions showing reliable network consistency differed between metrics revealed no significant differences (z-based test for comparing binomial distributions, all $P>0.06$). For amplitude-based networks, subject proportions

showing within-subject network consistency with the control metric were significantly lowered relative to the standard metric for 9 out of 12 comparisons, although, crucially, proportions of subjects exhibiting such consistency were still far greater than zero (40.9 ± 28.5% across all 12 comparisons).

Thus, while decreases in within-subject network consistency are apparent after the removal of volume conduction effects for amplitude-based networks, these findings importantly establish that the ability of phase- and amplitude-based connectivity patterns to distinguish between individuals is not lost when spurious connectivity induced by the same source projecting to nearby electrodes is removed.

Distinct rest and task network profiles across individuals

Assessing group-level differences of rest and task networks, wPLI-based analyses replicated the significant effects seen with standard phase synchrony, as reported in Supplementary Table 2A, for alpha and beta rest networks, but did not confirm the effects of theta rest and alpha task networks. In contrast, the control metric indicated theta task networks to be significantly more similar at the group-level than expected, a difference not seen for the standard phase synchrony metric. For amplitude-based connectivity, the control metric replicated the significant differences from the standard metric for theta and alpha rest networks. In addition, the control metric revealed above-chance similarity of group-level beta rest networks, an observation not made for the standard measure of amplitude connectivity. Thus, although some qualitative differences are apparent, these findings indicate that connectivity patterns defined by control metrics can be differentiated based on behavioral state.

To further support this idea, we performed classification analyses asking how accurately an out-of-fold subject's rest and task segments can be assigned the correct behavioral state. Performance using the control metrics was significantly reduced relative to standard connectivity metrics, except for amplitude-based gamma networks. Critically, however, we still achieved significantly ($P<0.05$, binomial test) above-chance (50%) performance rates for all phase-based networks and all amplitude-based networks except gamma (Supplementary Table 11).

Combined, these results indicate that neither phase- nor amplitude-based rest and task differences as reported in the main text can result solely from volume-conduction-based spurious connectivity.

Frequency-specific networks across individuals

Next, we examined if group-level networks can be differentiated based on frequency when using control connectivity metrics. For rest, wPLI-based results confirmed frequency-specificity for alpha, beta, and gamma networks, replicating the pattern of the standard metric for three out of four spectral bands seen in Supplementary Table 4A. For task segments, we confirmed the group-level separability of beta and gamma networks and, in addition, found theta networks to express group-level frequency-specificity, an observation not made for the standard metric. For amplitude-based networks, the control metric confirmed the results using

the standard metric for alpha and beta during rest, but not for theta and gamma during rest. For task, frequency-specific networks were replicated for beta but not gamma band, and were additionally found in the alpha band.

Thus, although some qualitative differences are again apparent, these results indicate that frequency-specific connectivity networks can be discerned at the group-level even when using metrics designed to minimize spurious connectivity.

Long-term stability of large-scale oscillatory networks

Finally, we assessed the long-term stability of subject-specific connectivity profiles by training classifiers on data segments from S_{AB} and testing them on S_C segments. Long-term subject recognition of data segments was significantly above chance (binomial, all $P<0.05$) for all network types, for both rest and task (Supplementary Table 12). For phase synchrony, the percentage of data segments assigned to the correct subject was closely comparable between the standard and control metric for most network types, and significantly increased for the control metric for alpha and beta rest networks. Amplitude-based subject recognition was generally lower for the control relative to the standard metric, although significant differences only emerged in the gamma band.

In sum, these findings indicate that our main results of long-term network stability cannot be attributed to spurious connectivity.

Interpretation

Given the preceding set of analyses, we conclude that the separation of oscillatory networks based on individual, behavioral state, and frequency, as well as long-term classification, is not simply due to spurious connectivity resulting from volume conduction. Specifically, although control connectivity measures lead to an overall reduction in both absolute connection strengths (for amplitude correlation; Supplementary Table 8) and the similarity of pairs of networks (for phase- and amplitude-based networks; Supplementary Table 9), the ability to differentiate between different network types and individuals remains intact when using these metrics. Although we did not repeat every possible analysis from the main text, we expect the present observations to generalize to other network comparisons (e.g., within-subject analyses, merging data segments and network types for classification).

What is unclear is whether the differences between standard and control metrics are entirely due to the removal of spurious, volume-conduction based effects. As noted above, some zero time lag synchrony does in fact reflect meaningful electrophysiological connectivity (Roelfsema et al., 1997; Slotnick et al., 2002; Stujenske et al., 2014). It is therefore not obvious which functional connectivity metrics are most appropriate for the analysis of large-scale EEG networks. Certainly for classifiers, the choice of tools should be based simply on which analytic methods yield the most successful classification performance, which appear to be the standard methods presented in the main text. Concerning network separability, our results also suggest that standard metrics, on aggregate, yield stronger evidence of network separation than control metrics. We speculate that this apparent improvement is at

least partly due to the availability of true zero-lag connectivity that assists in distinguishing between network types, and may therefore be preferred if the objective is to demonstrate such differences.

Supplementary Methods

Behavioral protocol

For S_A and S_B, each subject's sequence of blocks was organized around the encoding and retrieval of visuospatial associations of two distinct stimulus sets (Fig. 1). One set consisted of pictures of animals, the other of vehicles. During S_A, the first five blocks were organized as rest-encoding-rest-retrieval-rest, and pertained to the first stimulus category. Then, this sequence of blocks was repeated for the second stimulus set. The order of stimulus categories was counterbalanced across subjects (animals first: 11; vehicles first: 10). For reasons unrelated to the present report, on-screen instructions then informed participants that later, during S_B, they would be retested only on the first category they were trained on (see below). Then, a final S_A resting state recording was obtained. Thus, a total of 11 behavioral blocks took place, including 7 rest segments ($rest_{A1}$-$rest_{A7}$) and two encoding task segments ($task_{A1}$ and $task_{A2}$) for EEG analyses.

After a 2 h break, S_B began with a reminder that a retest would only be administered for the first encoding category. Then, three blocks were presented in the order rest-retrieval-rest, where the retrieval block reflected the first (and expected) stimulus category. This sequence was followed by a surprise notification that subjects would now also be tested on the second category, and a sequence of rest-retrieval-rest for that category ensued. Thus, for EEG analyses, S_B yielded 4 rest segments ($rest_{B1}$-$rest_{B4}$). An exit questionnaire probed subjects for their learning and retrieval strategies, the amount of time they spent thinking about the stimuli in different phases of the protocol, and their memory and beliefs concerning the expectancy manipulation.

The test expectancy manipulation was originally included to examine whether retest expectation would affect memory consolidation and, consequently, memory performance during S_B, and whether this might be reflected in the EEG. However, behavioral results did not provide any evidence for this hypothesis and we did not pursue this notion further. Importantly, this state of affairs does not provide a confounding influence hampering interpretation of our results. Beside the fact that memory was not affected by test expectation and we therefore think it unlikely a neural effect would be present, all our EEG analyses were performed across data segments irrespective of expectation category. Moreover, any effect of expectancy would presumably arise after the manipulation is introduced (or only after the surprise retest), and could therefore only affect segments $rest_{A7}$ and $rest_{B1}$-$rest_{B4}$ (or arguably only $rest_{B3}$-$rest_{B4}$). All in all, we do not believe this manipulation influences the interpretation of the presented analyses.

S_C consisted of one sequence of blocks organized as rest-encoding-rest-retrieval-rest, similar to S_A, and a sequence rest-control-rest. The order of these

sequences was counterbalanced across subjects (7 memory first, 7 control first). The stimulus set for memory included 18 animals and 18 vehicles from S_A. However, all subjects indicated they did not remember any picture-location pairs from their previous visit, 3 to 8 months earlier. In the control condition, a single stimulus picture was repeatedly presented at all locations, so no unique visuospatial memories could be formed. Thus, S_C provided rest segments $rest_{C1}$-$rest_{C5}$ and task segments $task_{C1}$ and $task_{C2}$ (one an encoding segment and the other a control segment) for EEG analysis.

Network permutations

Here, we offer a detailed account of our permutation analyses, organized by results section of the main text:

Network consistency within individuals: The consistency of within-subject network structure for each network type was determined, for rest segments, by selecting a subject's rest networks, computing all pair-wise correlations, and averaging them. For permutation testing, this procedure was repeatedly performed with shuffled subject labels (i.e., with segments selected randomly from across all subjects). Then, each subject's observed network similarity was compared to the permutation distribution as explained in the Methods. For task segments, the procedure was identical. For rest-task comparisons, we calculated all unique rest-task correlations for each subject. For permutation, subject labels of task segments were kept intact, but rest segment labels were repeatedly shuffled, producing pairs with a task segment from the individual under analysis, and a rest segment selected randomly from across subjects. As a result, a different null-distribution was generated for each subject.

Distinct rest and task network profiles across individuals: For each network type, we selected all task segments from all subjects, computed all pair-wise correlations, and averaged them. We did the same for rest, but using only two rest segments ($rest_{A2}$ and $rest_{A5}$) from each subject (to equalize the number of rest and task segments). For permutation testing, we repeatedly shuffled the labels of behavioral condition, and recalculated similarity values.

Frequency-specific networks for individuals: For every individual, oscillation metric, and behavioral state, we selected all networks of the same frequency, determined each pair-wise correlation, and averaged them. For permutation testing, we repeatedly shuffled frequency labels before recalculating similarity scores. For every individual, observed single-frequency network similarity in different frequency bands could then be compared to a baseline distribution of network similarity scores across frequency bands.

Frequency-specific networks across individuals: For every oscillation metric and behavioral state, we selected all networks across all subjects of the same frequency, and determined the average within-frequency network similarity. Frequency labels were then repeatedly shuffled to generate a surrogate distribution of group-level network similarity across frequencies. Observed group-level single-frequency similarity scores were then compared to this null distribution.

Distinct power-, phase-, and amplitude-based networks for individuals: For every individual, frequency band, and behavioral state, we selected all networks of the same oscillation metric. For power, we used the "power connectivity" values as explained in the Methods. We calculated every pair-wise correlation and averaged them to obtain an estimate of same-metric similarity. We then shuffled oscillation metric labels to generate a null distribution of cross-metric similarity, against which observed vales were compared.

Distinct power-, phase-, and amplitude-based networks across individuals: For every frequency band, and behavioral state, we selected all networks of the same oscillation metric across all subjects, and determined the average within-metric network similarity. Oscillatory metric labels were then repeatedly shuffled to generate a surrogate distribution of group-level network similarity across oscillation metrics.

Short-term/long-term network stability (Supplementary Results): For each network type, we calculated for each subject every unique correlation between segments from the to-be-compared sessions (rest-rest, task-task, rest-task, and task-rest, and for S_A-S_B and S_{AB}-S_C). For permutation testing, subject labels were kept intact for one session, but were repeatedly shuffled for the other one to generate a null distribution of similarity estimates.

Control connectivity metrics

For each frequency band, wPLI (Vinck et al., 2011) between every channel pair (j, k) was calculated as:

$$wPLI_{jk} = \frac{\frac{1}{n}\sum_{t=1}^{n} |imag(S_{jkt})| sgn(imag(S_{jkt}))}{\frac{1}{n}\sum_{t=1}^{n} |imag(S_{jkt})\,|}$$

where *imag* indicates the imaginary part, S_{jkt} is the cross-spectral density between signals *j* and *k* at sample *t*, and *sgn* indicates the sign. As for regular phase synchrony, wPLI was calculated for each of ten data fragments before averaging, neighboring connections were removed, and vectors were normalized using the box-cox transformation and z-scored.

For each frequency band, orthogonalization of amplitude envelopes (Hipp et al., 2012) was performed for each channel pair (j, k) such that:

$$j_{\perp k}(t) = \; imag(j(t)\frac{k(t)^*}{|k(t)|})$$

where *j(t)* and *k(t)* are complex-valued analytic signals derived from the Hilbert transform, and * indicates the complex conjugate. The orthogonalized envelope $j_{\perp k}(t)$ was then correlated with the envelope of signal k (i.e., $|k(t)|$) using the Spearman correlation. For each channel pair, orthogonalization and correlation was performed in both directions before averaging. Finally, values were averaged across data fragments, and resulting network vectors were normalized and z-scored.

Supplementary References

Hipp, J. F., Hawellek, D. J., Corbetta, M., Siegel, M., & Engel, A. K. (2012). Large-scale cortical correlation structure of spontaneous oscillatory activity. *Nature Neuroscience*, *15*(6), 884–890. https://doi.org/10.1038/nn.3101

Roelfsema, P. R., Engel, A. K., König, P., & Singer, W. (1997). Visuomotor integration is associated with zero time-lag synchronization among cortical areas. *Nature*, *385*(6612), 157–61. https://doi.org/10.1038/385157a0

Slotnick, S. D., Moo, L. R., Kraut, M. a, Lesser, R. P., & Hart, J. (2002). Interactions between thalamic and cortical rhythms during semantic memory recall in human. *Proceedings of the National Academy of Sciences of the United States of America*, *99*(9), 6440–6443. https://doi.org/10.1073/pnas.092514899

Stujenske, J. M., Likhtik, E., Topiwala, M. A., & Gordon, J. A. (2014). Fear and Safety Engage Competing Patterns of Theta-Gamma Coupling in the Basolateral Amygdala. *Neuron*, *83*(4), 919–933. https://doi.org/10.1016/j.neuron.2014.07.026

Vicente, R., Gollo, L. L., Mirasso, C. R., Fischer, I., & Pipa, G. (2008). Dynamical relaying can yield zero time lag neuronal synchrony despite long conduction delays. *Proceedings of the National Academy of Sciences*, *105*(44), 17157–17162. https://doi.org/10.1073/pnas.0809353105

Vinck, M., Oostenveld, R., Van Wingerden, M., Battaglia, F., & Pennartz, C. M. A. (2011). An improved index of phase-synchronization for electrophysiological data in the presence of volume-conduction, noise and sample-size bias. *NeuroImage*, *55*(4), 1548–1565. https://doi.org/10.1016/j.neuroimage.2011.01.055

Supplementary Tables

Supplementary Table 1A: Within-subject S_A network similarity, within and between behavioral states, for all oscillation metrics and frequency bands.

SESSION A		*observed mean ± SD*	*baseline mean*	*group t-test P value*	*individual permutation* % P_{corr}<0.05	*individual permutation* % P_{uncorr}<0.05
**rest A**						
phase-sync	*theta*	0.67 ± 0.10	0.31	$<10^{-13}$	100.0	100.0
	alpha	0.77 ± 0.10	0.28	$<10^{-15}$	100.0	100.0
	beta	0.72 ± 0.09	0.37	$<10^{-12}$	100.0	100.0
	gamma	0.64 ± 0.09	0.44	$<10^{-8}$	90.5	90.5
amp-corr	*theta*	0.57 ± 0.11	0.39	$<10^{-6}$	90.5	90.5
	alpha	0.75 ± 0.10	0.39	$<10^{-12}$	100.0	100.0
	beta	0.72 ± 0.09	0.48	$<10^{-9}$	95.2	95.2
	gamma	0.62 ± 0.09	0.44	$<10^{-8}$	90.5	90.5
power	*theta*	0.89 ± 0.06	0.54	$<10^{-16}$	100.0	100.0
	alpha	0.98 ± 0.03	0.84	$<10^{-15}$	95.2	95.2
	beta	0.84 ± 0.07	0.53	$<10^{-14}$	100.0	100.0
	gamma	0.79 ± 0.08	0.59	$<10^{-10}$	90.5	95.2
**task A**						
phase-sync	*theta*	0.59 ± 0.19	0.27	$<10^{-7}$	81.0	85.7
	alpha	0.64 ± 0.18	0.23	$<10^{-9}$	90.5	90.5
	beta	0.66 ± 0.14	0.37	$<10^{-8}$	85.7	85.7
	gamma	0.64 ± 0.14	0.43	$<10^{-6}$	61.9	81.0
amp-corr	*theta*	0.49 ± 0.16	0.27	$<10^{-5}$	66.7	76.2
	alpha	0.64 ± 0.17	0.33	$<10^{-7}$	76.2	81.0
	beta	0.69 ± 0.12	0.48	$<10^{-7}$	57.1	76.2
	gamma	0.67 ± 0.16	0.46	$<10^{-5}$	66.7	71.4
power	*theta*	0.80 ± 0.15	0.42	$<10^{-13}$	71.4	81.0
	alpha	0.86 ± 0.14	0.39	$<10^{-15}$	66.7	76.2
	beta	0.79 ± 0.13	0.45	$<10^{-12}$	0.0	61.9
	gamma	0.85 ± 0.09	0.64	$<10^{-8}$	0.0	52.4

Supplementary Table 1A (continued)

SESSION A		*observed* *mean ± SD*	*baseline* *mean*	*group t-test* *P value*	*individual permutation* *% P_{corr}<0.05*	*% P_{uncorr}<0.05*
**rest A - task A**						
phase-sync	*theta*	0.48 ± 0.11	0.25	$<10^{-8}$	100.0	100.0
	alpha	0.33 ± 0.17	0.15	$<10^{-4}$	81.0	85.7
	beta	0.49 ± 0.12	0.31	$<10^{-6}$	90.5	90.5
	gamma	0.54 ± 0.10	0.42	$<10^{-5}$	85.7	85.7
amp-corr	*theta*	0.39 ± 0.11	0.27	$<10^{-4}$	95.2	95.2
	alpha	0.39 ± 0.19	0.25	<0.002	66.7	66.7
	beta	0.57 ± 0.13	0.44	$<10^{-4}$	81.0	81.0
	gamma	0.54 ± 0.09	0.43	$<10^{-4}$	81.0	81.0
power	*theta*	0.67 ± 0.20	0.38	$<10^{-6}$	90.5	90.5
	alpha	0.70 ± 0.25	0.52	<0.002	95.2	100.0
	beta	0.53 ± 0.20	0.31	$<10^{-4}$	81.0	81.0
	gamma	0.73 ± 0.08	0.58	$<10^{-8}$	85.7	85.7

Observed: observed within-subject network similarity across subjects. Baseline: mean similarity estimates across permutations with shuffled subject labels (i.e., average between-subject similarity). Group t-test P value: significance level associated with one-sample t-test comparing observed values to baseline. Individual permutation, % P_{corr}<0.05: percentage of subjects with significant effects after correcting for multiple comparisons using False Discovery Rate; % P_{uncorr}<0.05: percentage of subjects below uncorrected permutation P value of 0.05. Based on n=21, 7 rest segments and 2 task segments.

Supplementary Table 1B: Within-subject S_A network similarity, within and between behavioral states, for all oscillation metrics and frequency bands, using only two rest segments.

SESSION A		*observed* *mean ± SD*	*baseline* *mean*	*group t-test* *P value*	*individual permutation* % P_{corr} < 0.05	*individual permutation* % P_{uncorr} < 0.05
**rest A**						
phase-sync	*theta*	0.67 ± 0.10	0.30	$<10^{-12}$	100.0	100.0
	alpha	0.77 ± 0.09	0.29	$<10^{-15}$	100.0	100.0
	beta	0.70 ± 0.10	0.38	$<10^{-11}$	90.5	95.2
	gamma	0.64 ± 0.11	0.44	$<10^{-7}$	61.9	66.7
amp-corr	*theta*	0.59 ± 0.10	0.39	$<10^{-7}$	47.6	61.9
	alpha	0.73 ± 0.12	0.42	$<10^{-10}$	52.4	66.7
	beta	0.73 ± 0.12	0.49	$<10^{-8}$	19.0	66.7
	gamma	0.62 ± 0.10	0.43	$<10^{-7}$	52.4	61.9
power	*theta*	0.91 ± 0.06	0.56	$<10^{-17}$	95.2	95.2
	alpha	0.98 ± 0.02	0.84	$<10^{-19}$	0.0	95.2
	beta	0.86 ± 0.10	0.52	$<10^{-12}$	0.0	81.0
	gamma	0.82 ± 0.12	0.59	$<10^{-7}$	0.0	57.1
**rest A-task A**						
phase-sync	*theta*	0.48 ± 012	0.25	$<10^{-7}$	95.2	100.0
	alpha	0.34 ± 0.15	0.16	$<10^{-4}$	71.4	76.2
	beta	0.51 ± 0.12	0.33	$<10^{-6}$	81.0	85.7
	gamma	0.55 ± 0.10	0.42	$<10^{-5}$	85.7	85.7
amp-corr	*theta*	0.40 ± 0.12	0.27	$<10^{-4}$	90.5	90.5
	alpha	0.40 ± 0.20	0.26	<0.003	47.6	52.4
	beta	0.57 ± 0.15	0.43	<0.001	61.9	61.9
	gamma	0.55 ± 0.10	0.43	$<10^{-4}$	57.1	61.9
power	*theta*	0.67 ± 0.20	0.38	$<10^{-6}$	85.7	85.7
	alpha	0.70 ± 0.25	0.52	<0.003	95.2	95.2
	beta	0.56 ± 0.20	0.32	$<10^{-4}$	52.4	57.1
	gamma	0.76 ± 0.09	0.59	$<10^{-7}$	61.9	61.9

Table structure similar to Supplementary Table 1A. Based on n=21, 2 rest segments ($rest_{A2}$ and $rest_{A5}$) and 2 task segments.

Supplementary Table 1C: Within-subject S_B network similarity of rest segments, for all oscillation metrics and frequency bands.

SESSION B		*observed mean ± SD*	*baseline mean*	*group t-test P value*	*individual permutation*	
					% P_{corr} < 0.05	*% P_{uncorr} < 0.05*
**rest B**						
phase-sync	*theta*	0.69 ± 0.14	0.28	$<10^{-11}$	100.0	100.0
	alpha	0.81 ± 0.12	0.24	$<10^{-14}$	100.0	100.0
	beta	0.74 ± 0.14	0.33	$<10^{-10}$	100.0	100.0
	gamma	0.65 ± 0.12	0.41	$<10^{-7}$	85.7	85.7
amp-corr	*theta*	0.60 ± 0.12	0.37	$<10^{-7}$	85.7	85.7
	alpha	0.76 ± 0.11	0.30	$<10^{-13}$	100.0	100.0
	beta	0.74 ± 0.11	0.44	$<10^{-10}$	95.2	95.2
	gamma	0.65 ± 0.12	0.43	$<10^{-7}$	81.0	81.0
power	*theta*	0.89 ± 0.07	0.49	$<10^{-16}$	100.0	100.0
	alpha	0.98 ± 0.02	0.83	$<10^{-20}$	100.0	100.0
	beta	0.88 ± 0.09	0.58	$<10^{-11}$	76.2	81.0
	gamma	0.83 ± 0.09	0.59	$<10^{-10}$	85.7	85.7

Table structure similar to Supplementary Table 1A. Based on n=21 and 4 rest segments.

Supplementary Table 1D: Within-subject S_C network similarity, within and between behavioral states, for all oscillation metrics and frequency bands.

SESSION C		*observed mean ± SD*	*baseline mean*	*group t-test P value*	*individual permutation* % P_{corr}<0.05	% P_{uncorr}<0.05
**rest C**						
phase-sync	*theta*	0.66 ± 0.11	0.28	$<10^{-8}$	100.0	100.0
	alpha	0.72 ± 0.16	0.26	$<10^{-7}$	92.9	92.9
	beta	0.71 ± 0.13	0.33	$<10^{-7}$	92.9	92.9
	gamma	0.65 ± 0.10	0.41	$<10^{-6}$	85.7	85.7
amp-corr	*theta*	0.57 ± 0.12	0.36	$<10^{-5}$	78.6	85.7
	alpha	0.71 ± 0.15	0.33	$<10^{-6}$	92.9	92.9
	beta	0.72 ± 0.12	0.44	$<10^{-6}$	92.9	92.9
	gamma	0.62 ± 0.11	0.41	$<10^{-5}$	78.6	85.7
power	*theta*	0.87 ± 0.10	0.52	$<10^{-8}$	92.9	92.9
	alpha	0.95 ± 0.09	0.78	$<10^{-5}$	0.0	85.7
	beta	0.81 ± 0.12	0.43	$<10^{-8}$	85.7	78.6
	gamma	0.81 ± 0.06	0.57	$<10^{-8}$	100.0	100.0
**task C**						
phase-sync	*theta*	0.55 ± 0.16	0.25	$<10^{-5}$	71.4	71.4
	alpha	0.59 ± 0.18	0.19	$<10^{-6}$	71.4	85.7
	beta	0.64 ± 0.14	0.33	$<10^{-5}$	71.4	78.6
	gamma	0.65 ± 0.14	0.40	$<10^{-5}$	64.3	71.4
amp-corr	*theta*	0.47 ± 0.12	0.27	$<10^{-4}$	50.0	57.1
	alpha	0.66 ± 0.18	0.34	$<10^{-5}$	71.4	78.6
	beta	0.69 ± 0.17	0.45	$<10^{-4}$	71.4	71.4
	gamma	0.67 ± 0.13	0.46	$<10^{-4}$	57.1	64.3
power	*theta*	0.84 ± 0.12	0.46	$<10^{-7}$	85.7	92.9
	alpha	0.90 ± 0.07	0.49	$<10^{-11}$	0.0	85.7
	beta	0.84 ± 0.14	0.44	$<10^{-7}$	0.0	85.7
	gamma	0.86 ± 0.09	0.65	$<10^{-6}$	0.0	42.9

Supplementary Table 1D (continued)

SESSION C		*observed* *mean ± SD*	*baseline* *mean*	*group t-test* *P value*	*individual permutation* *% P_{corr}<0.05*	*% P_{uncorr}<0.05*
**rest C-task C**						
phase-sync	*theta*	0.49 ± 0.11	0.25	$<10^{-5}$	100.0	100.0
	alpha	0.40 ± 0.13	0.18	$<10^{-4}$	92.9	92.9
	beta	0.51 ± 0.13	0.30	$<10^{-4}$	92.9	92.9
	gamma	0.52 ± 0.11	0.39	<0.001	92.9	92.9
amp-corr	*theta*	0.43 ± 0.08	0.28	$<10^{-5}$	100.0	100.0
	alpha	0.49 ± 0.17	0.31	<0.001	92.9	92.9
	beta	0.58 ± 0.16	0.42	<0.002	92.9	92.9
	gamma	0.54 ± 0.10	0.42	<0.001	85.7	85.7
power	*theta*	0.69 ± 0.13	0.41	$<10^{-5}$	92.9	92.9
	alpha	0.80 ± 0.23	0.61	<0.004	100.0	100.0
	beta	0.60 ± 0.18	0.33	$<10^{-4}$	78.6	78.6
	gamma	0.72 ± 0.08	0.58	$<10^{-5}$	71.4	71.4

Table structure similar to Supplementary Table 1A. Based on n=14, 5 rest segments and 2 task segments.

Supplementary Table 2A: Group-level S_A network similarity for rest and task segments, for all oscillation metrics and frequency bands.

SESSION A		*task*	*rest*	*baseline*	*classification (%)*
phase-sync	*theta*	0.27	**0.30***	0.27	**85.7****
	alpha	**0.23***	**0.29***	0.21	**88.1****
	beta	0.37	**0.38***	0.35	**83.3****
	gamma	0.43	0.44	0.43	**72.6****
amp-corr	*theta*	0.27	**0.39***	0.30	**82.1****
	alpha	0.33	**0.42***	0.32	**88.1****
	beta	0.48	0.49†	0.46	**83.3****
	gamma	0.46	0.43	0.44	**59.5****
power	*theta*	0.42	**0.56***	0.43	**73.8****
	alpha	0.39	**0.84***	0.56	**67.9****
	beta	**0.45***	**0.52***	0.40	**81.0****
	gamma	**0.64***	0.59	0.60	**59.5****

Based on n=21, 2 rest segments ($rest_{A2}$ and $rest_{A5}$) and 2 task segments. *: significantly greater than baseline similarity after correcting for multiple comparisons (2 behavioral states). **: significantly above chance (50%) classifier performance. †: beta amplitude correlation during rest significant at P_{uncorr}<0.05.

Supplementary Table 2B: Group-level S_C network similarity for rest and task segments, for all oscillation metrics and frequency bands.

**SESSION C**		*task*	*rest*	*baseline*	*classification (%)*
phase-sync	*theta*	0.25	0.27	0.26	**83.9****
	alpha	0.19	**0.24***	0.20	**69.6****
	beta	0.33	0.31	0.31	**67.9****
	gamma	0.4	0.38	0.39	**62.5****
amp-corr	*theta*	0.27	**0.36***	0.30	**69.6****
	alpha	0.34	0.34	0.33	**62.5****
	beta	0.45	0.42	0.43	**62.5****
	gamma	**0.46†**	0.40	0.43	55.4
power	*theta*	0.46	**0.52***	0.45	**76.8****
	alpha	0.49	**0.75***	0.61	55.4
	beta	**0.44***	0.39	0.38	**69.6****
	gamma	**0.65***	0.56	0.59	**66.1****

Based on n=14, 2 rest segments ($rest_{C2}$ and $rest_{C4}$) and 2 task segments. *: significantly greater than baseline similarity after correcting for multiple comparisons (2 behavioral states). **: significantly above chance (50%) classifier performance. †: gamma amplitude correlation during task significant at $P_{uncorr}<0.05$.

Supplementary Table 3A: Within-subject similarity across behavioral states vs. between-subject network similarity within behavioral states during S_A, for all oscillation metrics and frequency bands.

		rest-task within	*rest between*		*task between*	
**SESSION A**		*mean ± SD*	*mean ± SD*	*t-test P*	*mean ± SD*	*t-test P*
phase-sync	*theta*	0.48 ± 0.11	0.30 ± 0.04	**$<10^{-73}$**	0.42 ± 0.20	0.17
	alpha	0.33 ± 0.17	0.28 ± 0.04	**$<10^{-3}$**	0.39 ± 0.29	0.34
	beta	0.49 ± 0.12	0.38 ± 0.04	**$<10^{-29}$**	0.45 ± 0.22	0.39
	gamma	0.54 ± 0.10	0.45 ± 0.04	**$<10^{-27}$**	0.64 ± 0.15†	**<0.003**
amp-corr	*theta*	0.39 ± 0.11	0.38 ± 0.05	0.32	0.42 ± 0.20	0.63
	alpha	0.39 ± 0.19	0.39 ± 0.07	0.70	0.39 ± 0.29	0.90
	beta	0.57 ± 0.13	0.48 ± 0.05	**$<10^{-10}$**	0.45 ± 0.22	**<0.02**
	gamma	0.54 ± 0.09	0.44 ± 0.04	**$<10^{-26}$**	0.64 ± 0.15†	**<0.002**
power	*theta*	0.67 ± 0.20	0.54 ± 0.06	**$<10^{-18}$**	0.42 ± 0.20	**$<10^{-7}$**
	alpha	0.70 ± 0.25	0.84 ± 0.04†	**$<10^{-27}$**	0.39 ± 0.29	**$<10^{-5}$**
	beta	0.53 ± 0.20	0.53 ± 0.08	0.83	0.45 ± 0.22	0.09
	gamma	0.73 ± 0.08	0.58 ± 0.06	**$<10^{-28}$**	0.64 ± 0.15	**<0.01**

Within-subject network similarity across rest and task states was generally greater than between-subject similarity of networks. †: opposite relation (i.e., significantly greater group-level similarity vs. within-subject similarity). Based on n=21, 7 rest segments and 2 task segments.

Supplementary Table 3B: Within-subject similarity across behavioral states vs. between-subject network similarity within behavioral states during S_C, for all oscillation metrics and frequency bands.

SESSION C		*rest-task within*	*rest between*		*task between*	
		mean ± SD	*mean ± SD*	*t-test P*	*mean ± SD*	*t-test P*
phase-sync	*theta*	0.49 ± 0.11	0.28 ± 0.06	**$<10^{-36}$**	0.25 ± 0.12	**$<10^{-11}$**
	alpha	0.40 ± 0.13	0.26 ± 0.07	**$<10^{-12}$**	0.19 ± 0.14	**$<10^{-6}$**
	beta	0.51 ± 0.13	0.33 ± 0.08	**$<10^{-16}$**	0.33 ± 0.12	**$<10^{-6}$**
	gamma	0.52 ± 0.11	0.41 ± 0.06	**$<10^{-10}$**	0.40 ± 0.11	**<0.001**
amp-corr	*theta*	0.43 ± 0.08	0.36 ± 0.07	**$<10^{-4}$**	0.27 ± 0.11	**$<10^{-6}$**
	alpha	0.49 ± 0.17	0.34 ± 0.08	**$<10^{-10}$**	0.34 ± 0.18	**<0.002**
	beta	0.58 ± 0.16	0.44 ± 0.07	**$<10^{-10}$**	0.45 ± 0.15	**<0.003**
	gamma	0.54 ± 0.10	0.41 ± 0.06	**$<10^{-14}$**	0.46 ± 0.11	**<0.009**
power	*theta*	0.69 ± 0.13	0.52 ± 0.10	**$<10^{-9}$**	0.46 ± 0.16	**$<10^{-6}$**
	alpha	0.80 ± 0.23	0.78 ± 0.12	0.51	0.49 ± 0.32	**<0.001**
	beta	0.60 ± 0.18	0.42 ± 0.13	**$<10^{-6}$**	0.44 ± 0.23	**<0.02**
	gamma	0.72 ± 0.08	0.57 ± 0.07	**$<10^{-13}$**	0.65 ± 0.15	0.07

Table structure similar to Supplementary Table 3A. Based on n=14, 5 rest segments and 2 task segments.

Supplementary Table 4A: Frequency-specific networks at the individual and group level during S_A, for all oscillation metrics and behavioral states.

SESSION A							
**rest A**			*theta*	*alpha*	*beta*	*gamma*	*baseline*
phase-sync	indiv.	*similarity*	0.67 ± 0.10	0.77 ± 0.10	0.72 ± 0.09	0.64 ± 0.09	0.41 ± 0.06
		% P_{corr}<0.05	100.0	100.0	100.0	100.0	
	group	*similarity*	**0.30***	**0.28***	**0.37***	**0.45***	0.24
amp-corr	indiv.	*similarity*	0.57 ± 0.11	0.75 ± 0.10	0.72 ± 0.09	0.62 ± 0.09	0.44 ± 0.08
		% P_{corr}<0.05	71.4	100.0	100.0	85.7	
	group	*similarity*	**0.39***	**0.39***	**0.48***	**0.44***	0.32
power	indiv.	*similarity*	0.89 ± 0.06	0.98 ± 0.03	0.84 ± 0.07	0.79 ± 0.08	0.56 ± 0.08
		% P_{corr}<0.05	100.0	100.0	95.2	90.5	
	group	*similarity*	**0.54***	**0.84***	**0.53***	**0.58***	0.41
**task A**							
phase-sync	indiv.	*similarity*	0.59 ± 0.19	0.64 ± 0.18	0.66 ± 0.14	0.64 ± 0.14	0.44 ± 0.05
		% P_{uncorr}<0.05	19.0	19.0	38.1	33.3	
	group	*similarity*	0.27	0.23	**0.37***	**0.43***	0.26
amp-corr	indiv.	*similarity*	0.49 ± 0.16	0.64 ± 0.17	0.69 ± 0.12	0.67 ± 0.16	0.42 ± 0.07
		% P_{uncorr}<0.05	0.0	38.1	61.9	52.4	
	group	*similarity*	0.27	0.33	**0.48***	**0.46***	0.30
power	indiv.	*similarity*	0.80 ± 0.15	0.86 ± 0.14	0.79 ± 0.13	0.85 ± 0.09	0.52 ± 0.14
		% P_{uncorr}<0.05	33.3	47.6	9.5	23.8	
	group	*similarity*	**0.42***	**0.39***	**0.45***	**0.64***	0.31

Based on n=21, 7 rest segments and 2 task segments. * indicates significantly greater than baseline similarity after correcting for multiple comparisons (4 frequency bands). Note that for task segments, for analyses at the level of the individual, we report proportions of subjects meeting the *uncorrected* threshold. Given the limited amount of possible permutations, multiple comparison correction based on the False Discovery Rate was overly restrictive. See *Network similarity and statistics* in Methods.

Supplementary Table 4B: Frequency-specific networks at the individual and group level during S_B for all oscillation metrics.

**SESSION B**							
**rest B**			*theta*	*alpha*	*beta*	*gamma*	*baseline*
phase-sync	indiv.	*similarity*	0.69 ± 0.14	0.81 ± 0.12	0.74 ± 0.14	0.65 ± 0.12	0.41 ± 0.07
		% P_{corr}<0.05	90.5	100.0	95.2	85.7	
	group	*similarity*	**0.28***	**0.25***	**0.33***	**0.42***	0.22
amp-corr	indiv.	*similarity*	0.60 ± 0.12	0.76 ± 0.11	0.74 ± 0.11	0.65 ± 0.12	0.44 ± 0.08
		% P_{corr}<0.05	52.4	90.5	95.2	76.2	
	group	*similarity*	**0.37***	0.29	**0.44***	**0.43***	0.30
power	indiv.	*similarity*	0.89 ± 0.07	0.98 ± 0.02	0.88 ± 0.09	0.83 ± 0.09	0.55 ± 0.10
		% P_{corr}<0.05	76.2	95.2	85.7	57.1	
	group	*similarity*	**0.49***	**0.83***	**0.57***	**0.58***	0.40

Based on n=21 and 4 rest segments. * indicates significantly greater than baseline similarity after correcting for multiple comparisons (4 frequency bands).

Supplementary Table 4C: Frequency-specific networks at the individual and group level during S_C, for all oscillation metrics and behavioral states.

**SESSION C**							
**rest C**			*theta*	*alpha*	*beta*	*gamma*	*baseline*
phase-sync	indiv.	*similarity*	0.66 ± 0.11	0.72 ± 0.16	0.71 ± 0.13	0.65 ± 0.10	0.43 ± 0.05
		% P_{corr}<0.05	92.9	92.9	92.9	78.6	
	group	*similarity*	**0.29***	**0.26***	**0.33***	**0.41***	0.23
amp-corr	indiv.	*similarity*	0.57 ± 0.12	0.71 ± 0.15	0.72 ± 0.12	0.62 ± 0.11	0.44 ± 0.06
		% P_{corr}<0.05	35.7	85.7	92.9	64.3	
	group	*similarity*	**0.36***	**0.34***	**0.44***	**0.41***	0.30
power	indiv.	*similarity*	0.87 ± 0.10	0.95 ± 0.09	0.81 ± 0.12	0.81 ± 0.06	0.56 ± 0.11
		% P_{corr}<0.05	92.9	92.9	85.7	50.0	
	group	*similarity*	**0.51***	**0.78***	0.42	**0.57***	0.38
**task C**							
phase-sync	indiv.	*similarity*	0.55 ± 0.16	0.59 ± 0.18	0.64 ± 0.14	0.65 ± 0.14	0.44 ± 0.08
		% P_{uncorr}<0.05	0.0	21.4	42.9	42.9	
	group	*similarity*	0.25	0.19	**0.33***	**0.40***	0.24
amp-corr	indiv.	*similarity*	0.47 ± 0.12	0.66 ± 0.18	0.69 ± 0.17	0.67 ± 0.13	0.44 ± 0.10
		% P_{uncorr}<0.05	0.0	42.9	64.3	42.9	
	group	*similarity*	0.27	0.34	**0.45***	**0.46***	0.31
power	indiv.	*similarity*	0.84 ± 0.12	0.90 ± 0.07	0.84 ± 0.14	0.86 ± 0.09	0.53 ± 0.15
		% P_{uncorr}<0.05	28.6	64.3	7.1	28.6	
	group	*similarity*	**0.46***	**0.49***	**0.44***	**0.65***	0.32

Based on n=14, 5 rest segments and 2 task segments. * indicates significantly greater than baseline similarity after correcting for multiple comparisons (4 frequency bands). Note that for task segments, for analyses at the level of the individual, we report proportions of subjects meeting the *uncorrected* threshold. Given the limited amount of possible permutations, multiple comparison correction based on the False Discovery Rate was overly restrictive. See *Network similarity and statistics* in Methods.

Supplementary Table 5A: Oscillation metric-specific networks at the individual and group level during S_A, for all frequency bands and behavioral states.

SESSION A						
**rest A**			*phase-sync*	*amp-corr*	*power*	*baseline*
theta	indiv.	*similarity*	0.67 ± 0.10	0.57 ± 0.11	0.89 ± 0.06	0.34 ± 0.06
		% P_{corr}<0.05	100.0	100.0	100.0	
	group	*similarity*	**0.30***	**0.39***	**0.54 ***	0.21
alpha	indiv.	*similarity*	0.77 ± 0.10	0.75 ± 0.10	0.98 ± 0.03	0.55 ± 0.09
		% P_{corr}<0.05	95.2	90.5	100.0	
	group	*similarity*	0.28	**0.39***	**0.83***	0.36
beta	indiv.	*similarity*	0.72 ± 0.09	0.72 ± 0.09	0.84 ± 0.07	0.37 ± 0.08
		% P_{corr}<0.05	100.0	100.0	100.0	
	group	*similarity*	**0.37***	**0.48***	**0.52***	0.24
gamma	indiv.	*similarity*	0.64 ± 0.09	0.62 ± 0.09	0.79 ± 0.08	0.40 ± 0.06
		% P_{corr}<0.05	100.0	100.0	100.0	
	group	*similarity*	**0.45***	**0.44***	**0.57***	0.31
**task A**						
theta	group	*similarity*	**0.27***	**0.27***	**0.41***	0.18
alpha			**0.23***	**0.33***	**0.38***	0.15
beta			**0.37***	**0.48***	**0.44***	0.20
gamma			**0.43***	**0.46***	**0.63***	0.33

Based on n=21, 7 rest segments and 2 task segments. * indicates significantly greater than baseline similarity after correcting for multiple comparisons (3 oscillation metrics). Note that for task segments, given the severely limited number of possible permutations, we could not assess the existence of oscillation metric-specific networks at the individual level.

Supplementary Table 5B: Oscillation metric-specific networks at the individual and group level during S_B for all frequency bands.

**SESSION B**						
**rest B**			*phase-sync*	*amp-corr*	*power*	*baseline*
theta	indiv.	*similarity*	0.69 ± 0.14	0.60 ± 0.12	0.89 ± 0.08	0.35 ± 0.06
		% P_{corr}<0.05	95.2	85.7	100.0	
	group	*similarity*	**0.28***	**0.37***	**0.48***	0.20
alpha	indiv.	*similarity*	0.81 ± 0.12	0.76 ± 0.11	0.98 ± 0.02	0.53 ± 0.09
		% P_{corr}<0.05	90.5	90.5	100.0	
	group	*similarity*	0.25	0.29	**0.83***	0.31
beta	indiv.	*similarity*	0.74 ± 0.14	0.74 ± 0.11	0.87 ± 0.09	0.39 ± 0.08
		% P_{corr}<0.05	100.0	90.5	100.0	
	group	*similarity*	**0.33***	**0.44***	**0.57***	0.24
gamma	indiv.	*similarity*	0.65 ± 0.12	0.65 ± 0.12	0.82 ± 0.09	0.41 ± 0.07
		% P_{corr}<0.05	81.0	76.2	100.0	
	group	*similarity*	**0.42***	**0.43***	**0.57***	0.30

Based on n=21 and 4 rest segments. * indicates significantly greater than baseline after correcting for multiple comparisons (3 oscillation metrics).

Supplementary Table 5C: Oscillation metric-specific networks at the individual and group level during S_C, for all frequency bands and behavioral states.

**SESSION C**						
**rest C**			*phase-sync*	*amp-corr*	*power*	*baseline*
theta	indiv.	*similarity*	0.66 ± 0.11	0.57 ± 0.12	0.87 ± 0.10	0.32 ± 0.04
		% P_{corr}<0.05	92.9	85.7	100.0	
	group	*similarity*	**0.29***	**0.36***	**0.51***	0.18
alpha	indiv.	*similarity*	0.72 ± 0.16	0.71 ± 0.15	0.95 ± 0.09	0.49 ± 0.11
		% P_{corr}<0.05	85.7	78.6	100.0	
	group	*similarity*	0.26	0.34	**0.77***	0.30
beta	indiv.	*similarity*	0.71 ± 0.13	0.72 ± 0.12	0.81 ± 0.12	0.35 ± 0.11
		% P_{corr}<0.05	92.9	92.9	100.0	
	group	*similarity*	**0.33***	**0.44***	**0.42***	0.18
gamma	indiv.	*similarity*	0.65 ± 0.10	0.62 ± 0.11	0.80 ± 0.06	0.39 ± 0.06
		% P_{corr}<0.05	92.9	78.6	100.0	
	group	*similarity*	**0.41***	**0.41***	**0.56***	0.28
**task C**						
theta	group	*similarity*	**0.25***	**0.27***	**0.45***	0.17
alpha			0.19	**0.34***	**0.49***	0.18
beta			**0.33***	**0.45***	**0.44***	0.18
gamma			**0.40***	**0.46***	**0.64***	0.32

Based on n=14, 5 rest segments and 2 task segments. * indicates significantly greater than baseline similarity after correcting for multiple comparisons (3 oscillation metrics). Note that for task segments, given the severely limited number of possible permutations, we could not assess the existence of oscillation metric-specific networks at the individual level.

Supplementary Table 5D: Connectivity metric-specific networks at the individual and group level during S_A, for all frequency bands and behavioral states.

SESSION A					
**rest A**			*phase-sync*	*amp-corr*	*baseline*
theta	indiv.	*similarity*	0.67 ± 0.10	0.57 ± 0.11	0.54 ± 0.08
		% P_{corr}<0.05	90.5	33.3	
	group	*similarity*	0.30	**0.39***	0.31
alpha	indiv.	*similarity*	0.77 ± 0.10	0.75 ± 0.10	0.68 ±0.09
		% P_{corr}<0.05	81.0	47.6	
	group	*similarity*	0.28	**0.39***	0.31
beta	indiv.	*similarity*	0.72 ± 0.09	0.72 ± 0.09	0.61 ± 0.07
		% P_{corr}<0.05	76.2	81.0	
	group	*similarity*	0.37	**0.48***	0.38
gamma	indiv.	*similarity*	0.64 ± 0.09	0.62 ± 0.09	0.60 ± 0.06
		% P_{corr}<0.05	47.6	19.0	
	group	*similarity*	**0.45***	**0.44***	0.43
**task A**					
theta	group	*similarity*	0.27	0.27	0.26
alpha			0.23	**0.33***	0.25
beta			0.37	**0.48***	0.39
gamma			0.43	**0.46***	0.43

Based on n=21, 7 rest segments and 2 task segments. * indicates significantly greater than baseline similarity after correcting for multiple comparisons (2 oscillation metrics). Note that for task segments, given the severely limited number of possible permutations, we could not assess the existence of oscillation metric-specific networks at the individual level.

Supplementary Table 5E: Connectivity metric-specific networks at the individual and group level during S_B for all frequency bands.

**SESSION B**					
**rest B**			*phase-sync*	*amp-corr*	*baseline*
theta	indiv.	*similarity*	0.69 ± 0.14	0.60 ± 0.12	0.57 ± 0.09
		% P_{corr}<0.05	81.0	28.6	
	group	*similarity*	0.28	**0.37***	0.30
alpha	indiv.	*similarity*	0.81 ± 0.12	0.76 ± 0.11	0.72 ± 0.11
		% P_{corr}<0.05	76.2	33.3	
	group	*similarity*	0.25	**0.29***	0.26
beta	indiv.	*similarity*	0.74 ± 0.14	0.74 ± 0.11	0.65 ± 0.09
		% P_{corr}<0.05	66.7	66.7	
	group	*similarity*	0.33	**0.44***	0.35
gamma	indiv.	*similarity*	0.65 ± 0.12	0.65 ± 0.12	0.63 ± 0.09
		% P_{corr}<0.05	19.0	19.0	
	group	*similarity*	0.42	**0.43***	0.42

Based on n=21 and 4 rest segments. * indicates significantly greater than baseline similarity after correcting for multiple comparisons (2 oscillation metrics).

Supplementary Table 5F: Connectivity metric-specific networks at the individual and group level during S_C, for all frequency bands and behavioral states.

**SESSION C**					
**rest C**			*phase-sync*	*amp-corr*	*baseline*
theta	indiv.	*similarity*	0.66 ± 0.11	0.57 ± 0.12	0.54 ± 0.08
		% P_{corr}<0.05	85.7	35.7	
	group	*similarity*	0.29	**0.36***	0.27
alpha	indiv.	*similarity*	0.72 ± 0.16	0.71 ± 0.15	0.64 ± 0.12
		% P_{corr}<0.05	57.1	50.0	
	group	*similarity*	0.26	**0.34***	0.27
beta	indiv.	*similarity*	0.71 ± 0.13	0.72 ± 0.12	0.59 ± 0.10
		% P_{corr}<0.05	92.9	78.6	
	group	*similarity*	0.33	**0.44***	0.33
gamma	indiv.	*similarity*	0.65 ± 0.10	0.62 ± 0.11	0.61 ± 0.06
		% P_{corr}<0.05	50.0	14.3	
	group	*similarity*	0.41	0.41	0.40
task C					
theta	group	*similarity*	0.25	0.27	0.25
alpha			0.19	**0.34***	0.24
beta			0.33	**0.45***	0.35
gamma			0.40	**0.46***	0.42

Based on n=14, 5 rest segments and 2 task segments. * indicates significantly greater than baseline similarity after correcting for multiple comparisons (2 oscillation metrics). Note that for task segments, given the severely limited number of possible permutations, we could not assess the existence of oscillation metric-specific networks at the individual level.

Supplementary Table 6A: Within-subject network similarity between S_A and S_B, for all oscillation metrics, frequency bands and behavioral states.

**SESSIONS A-B**		*observed*	*baseline*	*group t-test*	*individual permutation*	
**rest A-rest B**		*mean ± SD*	*mean*	*P value*	*% P_{corr}<0.05*	*% P_{uncorr}<0.05*
phase-sync	*theta*	0.56 ± 0.13	0.29	$<10^{-8}$	100.0	100.0
	alpha	0.68 ± 0.12	0.26	$<10^{-12}$	100.0	100.0
	beta	0.64 ± 0.13	0.35	$<10^{-9}$	100.0	100.0
	gamma	0.60 ± 0.11	0.43	$<10^{-6}$	90.5	90.5
amp-corr	*theta*	0.53 ± 0.13	0.38	$<10^{-4}$	95.2	95.2
	alpha	0.67 ± 0.13	0.33	$<10^{-10}$	100.0	100.0
	beta	0.66 ± 0.12	0.45	$<10^{-6}$	100.0	100.0
	gamma	0.58 ± 0.10	0.44	$<10^{-5}$	95.2	95.2
power	*theta*	0.81 ± 0.09	0.51	$<10^{-12}$	100.0	100.0
	alpha	0.96 ± 0.05	0.84	$<10^{-9}$	100.0	100.0
	beta	0.80 ± 0.09	0.55	$<10^{-10}$	95.2	95.2
	gamma	0.78 ± 0.08	0.58	$<10^{-10}$	95.2	95.2
**task A-rest B**						
phase-sync	*theta*	0.40 ± 0.14	0.24	$<10^{-4}$	90.5	90.5
	alpha	0.28 ± 0.18	0.13	<0.001	57.1	61.9
	beta	0.42 ± 0.15	0.28	<0.001	76.2	76.2
	gamma	0.51 ± 0.12	0.41	<0.001	61.9	71.4
amp-corr	*theta*	0.37 ± 0.13	0.27	<0.001	76.2	81.0
	alpha	0.35 ± 0.20	0.22	<0.005	47.6	47.6
	beta	0.55 ± 0.15	0.43	<0.001	61.9	66.7
	gamma	0.52 ± 0.11	0.43	<0.001	61.9	66.7
power	*theta*	0.61 ± 0.20	0.37	$<10^{-4}$	85.7	85.7
	alpha	0.68 ± 0.26	0.52	<0.005	100.0	100.0
	beta	0.51 ± 0.18	0.32	$<10^{-4}$	61.9	61.9
	gamma	0.73 ± 0.08	0.58	$<10^{-7}$	52.4	57.1

Observed similarity scores (mean ± SD) across subjects. Baseline: mean similarity score across permutations. Group t-test P value: significance level associated with one-sample t-test comparing observed values to baseline. Individual permutation, % P_{corr}<0.05: percentage of subjects with significant effects after correcting for multiple comparisons using False Discovery Rate; % P_{uncorr}<0.05: percentage of subjects below uncorrected permutation P value of 0.05. Based on n=21, 7 rest A and 4 rest B segments, and 2 task A segments.

Supplementary Table 6B: Intra-individual network similarity between S_{AB} and S_C within behavioral states, for all oscillation metrics and frequency bands.

**SESSIONS AB-C**		*observed*	*baseline*	*group t-test*	*individual permutation*	
**rest AB-rest C**		*mean ± SD*	*mean*	*P value*	*% P_{corr}<0.05*	*% P_{uncorr}<0.05*
phase-sync	*theta*	0.39 ± 0.11	0.28	<0.002	92.9	92.9
	alpha	0.43 ± 0.14	0.25	<0.001	92.9	92.9
	beta	0.47 ± 0.12	0.33	<0.001	92.9	92.9
	gamma	0.50 ± 0.11	0.41	<0.01	71.4	71.4
amp-corr	*theta*	0.45 ± 0.14	0.36	<0.02	85.7	85.7
	alpha	0.47 ± 0.16	0.32	<0.01	71.4	71.4
	beta	0.56 ± 0.13	0.44	<0.01	78.6	78.6
	gamma	0.52 ± 0.11	0.42	<0.01	85.7	85.7
power	*theta*	0.70 ± 0.13	0.50	$<10^{-4}$	92.9	92.9
	alpha	0.90 ± 0.09	0.80	<0.001	100.0	100.0
	beta	0.67 ± 0.13	0.46	$<10^{-4}$	100.0	100.0
	gamma	0.72 ± 0.08	0.58	$<10^{-5}$	100.0	100.0
**task A-task C**						
phase-sync	*theta*	0.36 ± 0.12	0.26	<0.01	78.6	78.6
	alpha	0.42 ± 0.15	0.21	$<10^{-4}$	92.9	92.9
	beta	0.47 ± 0.11	0.35	<0.001	78.6	78.6
	gamma	0.52 ± 0.12	0.41	<0.01	50.0	64.3
amp-corr	*theta*	0.36 ± 0.12	0.27	<0.01	57.1	64.3
	alpha	0.55 ± 0.19	0.33	<0.001	85.7	85.7
	beta	0.58 ± 0.15	0.46	<0.01	28.6	57.1
	gamma	0.59 ± 0.09	0.47	<0.001	64.3	64.3
power	*theta*	0.62 ± 0.14	0.45	<0.001	42.9	64.3
	alpha	0.77 ± 0.11	0.42	$<10^{-7}$	42.9	78.6
	beta	0.69 ± 0.13	0.46	$<10^{-5}$	50.0	64.3
	gamma	0.79 ± 0.11	0.65	<0.001	42.9	42.9

Table structure similar to Supplementary Table 6A. Based on n=14, 7 rest A, 4 rest B and 5 rest C segments, and 2 task A and 2 task C segments.

Supplementary Table 6C: Intra-individual network similarity between S_{AB} and S_C across behavioral states, for all oscillation metrics and frequency bands.

**SESSIONS AB-C**		*observed*	*baseline*	*group t-test*	*individual permutation*	
**rest AB-task C**		*mean ± SD*	*mean*	*P value*	*% P_{corr}<0.05*	*% P_{uncorr}<0.05*
phase-sync	*theta*	0.33 ± 0.11	0.24	<0.01	85.7	85.7
	alpha	0.27 ± 0.15	0.16	<0.02	71.4	71.4
	beta	0.37 ± 0.11	0.30	<0.02	71.4	71.4
	gamma	0 45 ± 0.11	0.39	<0.04	50.0	50.0
amp-corr	*theta*	0.34 ± 0.10	0.27	<0.02	78.6	78.6
	alpha	0.39 ± 0.18	0.29	<0.03	57.1	64.3
	beta	0.52 ± 0.14	0.44	<0.02	71.4	71.4
	gamma	0.48 ± 0.10	0.43	<0.03	57.1	57.1
power	*theta*	0.57 ± 0.15	0.40	<0.001	78.6	85.7
	alpha	0.72 ± 0.24	0.61	<0.05	100.0	100.0
	beta	0.46 ± 0.17	0.31	<0.01	57.1	64.3
	gamma	0.65 ± 0.10	0.56	<0.01	42.9	50.0
**task A-rest C**						
phase-sync	*theta*	0.30 ± 0.09	0.24	<0.02	71.4	71.4
	alpha	0.25 ± 0.14	0.16	<0.02	50.0	50.0
	beta	0.37 ± 0.12	0.31	<0.03	42.9	50.0
	gamma	0.46 ± 0.13	0.40	<0.05	64.3	64.3
amp-corr	*theta*	0.31 ± 0.12	0.25	<0.06	42.9	50.0
	alpha	0.36 ± 0.20	0.27	<0.05	50.0	57.1
	beta	0.49 ± 0.15	0.42	<0.06	50.0	64.3
	gamma	0.48 ± 0.10	0.42	<0.04	42.9	42.9
power	*theta*	0.50 ± 0.21	0.35	<0.02	64.3	64.3
	alpha	0.67 ± 0.25	0.50	<0.02	92.9	92.9
	beta	0.48 ± 0.17	0.33	<0.01	50.0	50.0
	gamma	0.69 ± 0.10	0.59	<0.001	57.1	64.3

Table structure similar to Supplementary Table 6A. Based on n=14, 7 rest A, 4 rest B and 5 rest C segments, and 2 task A and 2 task C segments.

Supplementary Table 7: Classifier performance for all oscillation metrics and frequency bands during rest, based on two rest segments per subject.

**rest A-rest C**	*theta*	*alpha*	*beta*	*gamma*
phase-sync	53.6	57.1	50.0	28.6
amp-corr	25.0	46.4	28.6	28.6
power	46.4	53.6	32.1	28.6

Numbers indicate percentage of data segments correctly identified. All classifiers performed significantly above chance (4.8%).

Supplementary Table 8: Comparison of raw connectivity estimates between standard and control metrics for an example subject's $rest_{A1}$ network.

	phase synchrony			*amplitude correlation*		
	standard	*control*	*paired t test*	*standard*	*control*	*paired t test*
theta	0.11 ± 0.05	0.10 ± 0.03	0.17	0.05 ± 0.03	0.00 ± 0.03	$<10^{-100}$
alpha	0.17 ± 0.08	0.19 ± 0.09	$<10^{-23}$	0.12 ± 0.05	0.00 ± 0.01	$<10^{-100}$
beta	0.093 ± 0.06	0.088 ± 0.04	$<10^{-4}$	0.12 ± 0.05	0.00 ± 0.01	$<10^{-100}$
gamma	0.09 ± 0.07	0.05 ± 0.03	$<10^{-112}$	0.03 ± 0.03	0.00 ± 0.00	$<10^{-100}$

Supplementary Table 9: Comparison of network similarity between standard and control metrics for an example subject's $rest_{A1}$-$rest_{A2}$ network pair.

	phase synchrony		*amplitude correlation*	
	standard	*control*	*standard*	*control*
theta	0.74	0.35	0.57	0.00
alpha	0.87	0.70	0.72	0.18
beta	0.81	0.55	0.82	0.17
gamma	0.66	0.28	0.57	0.08

Supplementary Table 10: Subject percentages with significantly above-chance within-subject network similarity for standard and control metrics.

		phase synchrony			*amplitude correlation*		
		standard	*control*	*z test*	*standard*	*control*	*z test*
rest	*theta*	100.0	90.5	0.15	90.5	33.3	$<10^{-3}$
	alpha	100.0	100.0	1	100.0	95.2	0.31
	beta	100.0	95.2	0.31	95.2	71.4	0.04
	gamma	90.5	95.2	0.55	90.5	28.6	$<10^{-4}$
task	*theta*	81.0	85.7	0.68	66.7	0	$<10^{-5}$
	alpha	90.5	90.5	1	76.2	42.9	0.03
	beta	85.7	71.4	0.26	57.1	38.1	0.22
	gamma	61.9	42.9	0.22	66.1	19.0	0.002
rest-task	*theta*	100.0	85.7	0.07	95.2	4.8	$<10^{-8}$
	alpha	81.0	76.2	0.71	66.7	76.2	0.49
	beta	90.5	71.4	0.12	81.0	47.7	0.02
	gamma	85.7	81.0	0.68	81.0	33.3	0.002

Supplementary Table 11: Correct classification percentages of segments' behavioral state for standard and control metrics.

	phase synchrony			*amplitude correlation*		
	standard	*control*	*z test*	*standard*	*control*	*z test*
theta	85.7	69.0	0.01	82.1	60.7	0.002
alpha	88.1	73.8	0.02	88.1	70.2	0.004
beta	83.3	61.9	0.002	83.3	65.5	0.008
gamma	72.6	58.3	0.05	59.5	50.0	0.21

Supplementary Table 12: Long-term subject classification of data segments for standard and control metrics.

		phase synchrony			*amplitude correlation*		
		standard	*control*	*z test*	*standard*	*control*	*z test*
rest	*theta*	58.6	45.7	0.13	32.9	21.4	0.13
	alpha	72.9	92.9	0.002	62.9	54.3	0.30
	beta	67.1	82.9	0.03	62.9	47.1	0.06
	gamma	47.1	44.3	0.73	50.0	20.0	$<10^{-3}$
task	*theta*	53.6	50.0	0.79	35.7	14.3	0.06
	alpha	78.6	67.9	0.37	71.4	50.0	0.10
	beta	57.1	60.7	0.79	39.3	35.7	0.78
	gamma	42.9	46.4	0.79	57.1	10.7	$<10^{-3}$
